# Side-chain Modifications of Highly Functionalized 3(2*H*)-Furanones

**DOI:** 10.3390/molecules171012151

**Published:** 2012-10-16

**Authors:** Viviani Nardini, Shirley Muniz Machado Rodrigues, Maurício Gomes Constantino, Gil Valdo José da Silva

**Affiliations:** Departamento de Química, Faculdade de Filosofia, Ciências e Letras de Ribeirão Preto, Universidade de São Paulo, Avenida dos Bandeirantes, 3900, 14040-901 Ribeirão Preto, SP, Brazil

**Keywords:** 3(2*H*)-furanone, isoxazole, natural products, side-chain modifications

## Abstract

A series of 3(*2H*)-furanones, based on side-chain modifications of a parent 3(*2H*)-furanone, was synthesized in good yield. The parent compound was prepared by hydrogenolysis, and subsequent acid hydrolysis, of isoxazole derivatives. The isoxazole was prepared by a [3+2] 1,3-dipolar cycloaddition reaction between 3-butyn-2-ol and nitrile oxide.

## 1. Introduction

**Figure 1:**
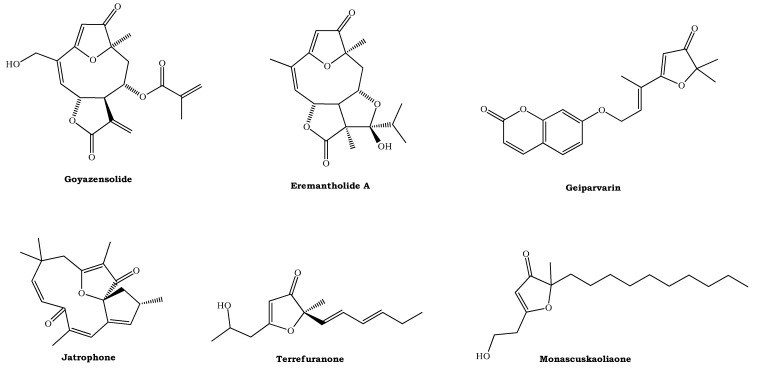
Examples of natural products containing the 3(2*H*)-furanone unit.

As a prominent structural feature, 3(2*H*)-furanone seems to be relevant in many classes of biologically active natural products, such as the examples collected in Figure 1 [1,2,3,4]. Sesquiterpene lactones encompass a large class of natural products showing a large diversity of molecular structures and a variety of biological activities. For a long time, much attention has been given to the α-methylenelactone unit, as can be seen in the structure of goyazensolide, because this group is considered to be responsible for activities such as cytotoxicity [5,6], through a mechanism involving a Michael addition of a cysteine sulfhydryl group [7]. Later, it was shown that many sesquiterpene lactones lacking the α-methylene group in the lactone unit, such as the eremantholides, present the same sort of activity. It seems reasonable that the 3(*2H*)-furanone unit could be the electrophilic center responsible for the activity of eremantholides [5,6]. A large number of biological active natural compounds possessing the 3(2*H*)-furanone unit have been isolated. Geiparvarin and jatrophone, for example, have antitumor activity [8,9]. 

In recent years, several synthetic methods have been applied to build these skeletons [10,11]. Chimichi and co-wokers [8,9] reported the preparation of 3(2*H*)-furanones through hydrogenolysis and subsequent acid hydrolysis of isoxazole derivatives. This approach is very interesting because it allows the preparation of series of 3(2*H*)-furanones possessing different side chains, which may also contain reactive functional groups. Such compounds are useful for studies related to biological activities of furanones and as starting material for the synthesis of sesquiterpene lactones such as furanoheliangolides.

Since we are interested both in the preparation of libraries of compounds for biological testing and in the development of synthetic methodology for the preparation of natural products, we disclose herein our exploration of Chimichi’s method for the preparation of a series of structurally modified 3(2*H*)-furanones.

## 2. Results and Discussion

**Scheme 1:**
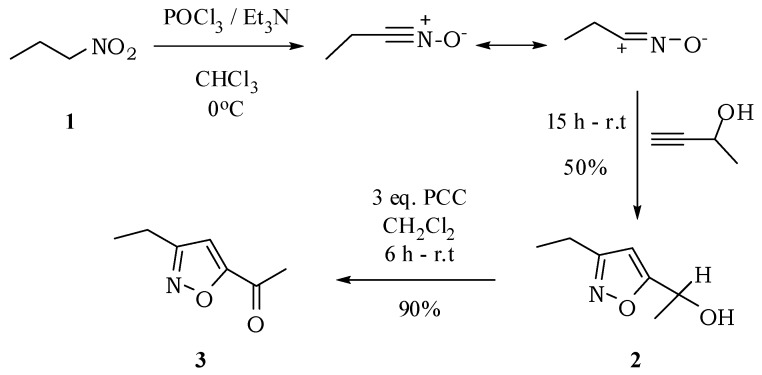
Preparation of keto-isoxazole **3**.

Following Chimichi’s procedure, isoxazole **2** was prepared by a [3+2] 1,3-dipolar cycloaddition reaction between 3-butyn-2-ol and nitrile oxide, prepared *in situ* from nitropropane (Scheme 1) [8]. PCC oxidation of compound **2** furnished the keto-isoxazole **3** in good yield. In the original paper the authors prepared ketone **3** by an alternate route, by MnO_2_ oxidation of the isoxazoline obtained from nitrile oxide and methyl vinyl ketone.

**Scheme 2:**
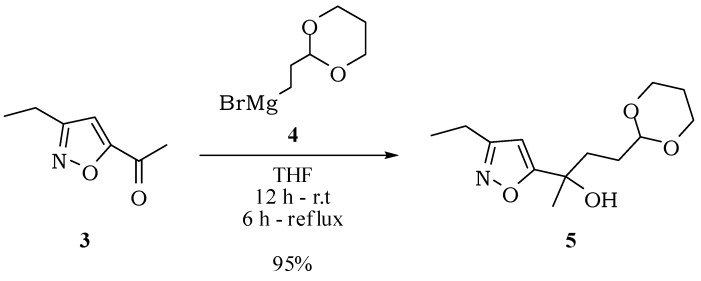
Preparation of compound **5**.

Ketone **3** is a key intermediate to introduce a suitable side chain into the future furanone ring. For the intended series of compounds, we choose to treat compound **3** with the Grignard reagent **4**, which contains a protected aldehyde as a reactive functional group for future use. The reagent **4** was prepared from commercially available 2-(2-bromoethyl)-1,3-dioxane in THF [12], affording **5** in 95% yield (Scheme 2). 

**Scheme 3:**
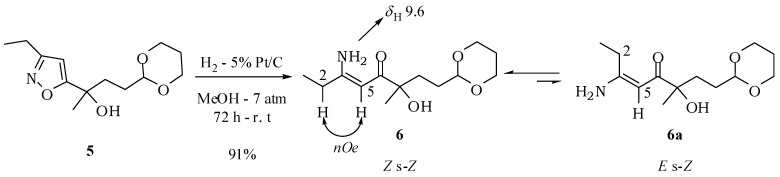
Hydrogenolysis of isoxazole **5**.

Hydrogenolysis of isoxazoles followed by acid hydrolysis leads to furanones [8]. Thus, compound **5** was exposed to a hydrogen atmosphere in the presence of platinum supported on carbon leading to the β-aminoenone **6** in 91% yield. It is interesting to note that β-aminoenones can usually occur as two isomers **6** and **6a**, which can be distinguished from each other by typical H(N) chemical shifts (*E*-isomer: *δ*_H_ 4.0–8.0; *Z*-isomer: *δ*_H_ 9.0–13.0) [13]. In our case, we have obtained only one isomer; structure **6** was assigned to this isomer based both on the H(N) chemical shift (*δ*_H_ 9.6) and on the observation of a nOe effect between H2 and H5 (Scheme 3).

**Figure 2:**
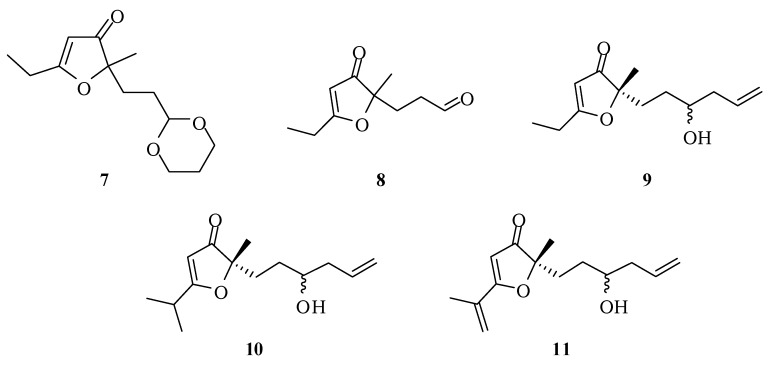
The series of 3(2*H*)-furanones prepared in this work.

Compound **6** is the precursor of the planned series of 3(*2H*)-furanones. We report here the preparation of the compounds depicted in Figure 2. This series can be easily expanded since the side chain is suitably functionalized.

**Scheme 4:**
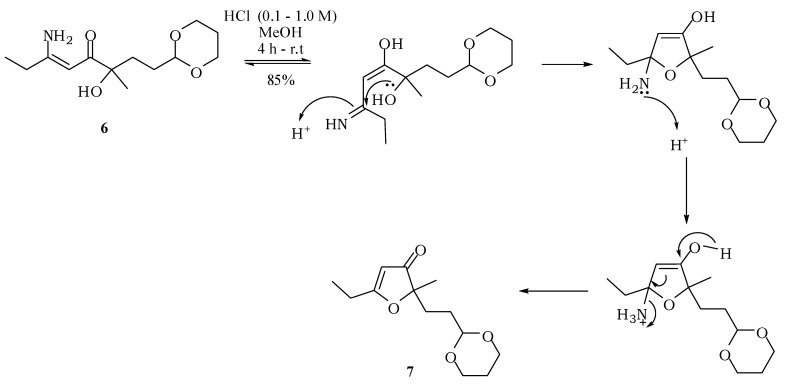
Proposed mechanism for the cyclization of β-aminoenone.

The parent compound of the series, **7**, was prepared in 85% yield from **6** by treatment with hydrochloric acid. Under mild acidic conditions, the enol-imine tautomeric form of **6** provides a favorable *5-exo-trig* arrangement for the cyclization in accordance with Baldwin’s rules (Scheme 4) [14].

**Scheme 5:**
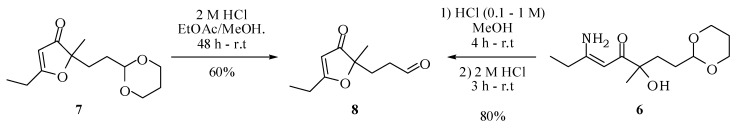
Two procedures to obtain furanone **8**.

The second furanone in the series, **8**, was obtained in 60% yield by treatment of **7** with more concentrated hydrochloric acid (2 M). Later, compound **8** was obtained in 80% yield directly from **6** using a “one pot” procedure, starting with the treatment of compound **6** with 0.1 M HCl and three subsequent additions of 1 M HCl in one-hour intervals to speed up the cyclization of β-aminoenone **6**, and then, 2 M HCl was added to hydrolyze the acetal (Scheme 5).

**Scheme 6:**
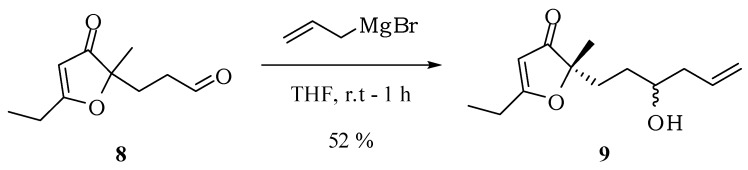
Elongation of side the chain of compound **8**.

At this point, we elongated the side chain of compound **8** with allylmagnesium bromide [15] (Scheme 6) obtaining compound **9** in 52% yield. The product seems to be homogenous either by TLC and column chromatography, however the ^13^C-NMR spectrum shows duplicates for seven out of the expected 14 signals, suggesting the formation of two diastereoisomers. In the ^1^H-NMR spectrum, only the signal of the coupled methyl group appears as a duplicated triplet, and all other signals of both diastereoisomers remained unresolved. All attempts to separate the two diastereoisomers by TLC were unsuccessful. The relative intensities of the two triplets mentioned above indicate that the diastereoselectivity of the addition of the allylmagnesium bromide to the aldehyde **8** was very poor, leading to almost equivalent amounts of both isomers.

**Scheme 7:**
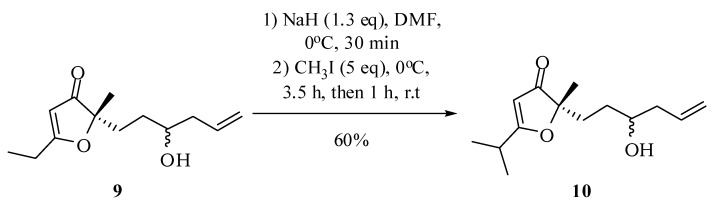
Alkylation of compound **9**.

An alkylation reaction is all that is needed to prepare compound **10** from compound **9**. Treatment of **9** with NaH in THF should lead to an equilibrium mixture of the alkoxide and the enolate of the unsaturated ketone. The addition of CH_3_I to this mixture, however, resulted only in the C-alkylation product, thus transforming the ethyl into an isopropyl substituent, giving rise to **10** with 60% yield (Scheme 7). 

**Scheme 8:**
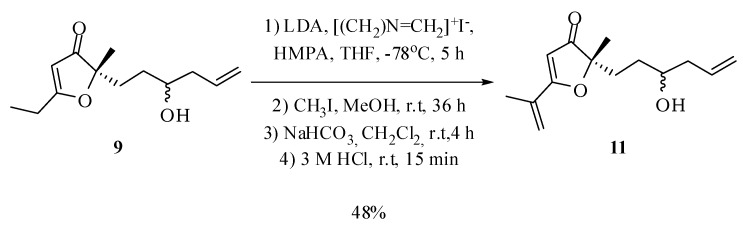
Introduction of a methylene group in compound **9**.

Finally, the ethyl group of compound **9** was easily transformed into an isopropylidene group through an adaptation of the Danishefsky’s method [16,17], originally developed to insert a methylene group in the α position of a lactone, affording the last compound in Figure 2, furanone **11** (Scheme 8).

As occurred with compound **9**, all attempts to separate the diastereoisomers of **10** and **11** were unsuccessful. 

## 3. Experimental

### 3.1. General 

Melting points were determined on a Kofler hot plate with an uncalibrated thermometer, installed on a Bristoline microscope. The purification of reaction products was performed by column chromatography using silica gel (70–230 mesh). Analytical thin-layer chromatography was performed on silica gel 60 F_254_ aluminum sheets. Visualization was accomplished with UV light and vanillin solution followed by heating. The infrared spectra were obtained in a Perkin-Elmer Spectrum RX IFTIR System. The wavelengths of maximum absorbance (max) are quoted in wavenumbers (cm^−1^). ^1^H and proton-decoupled ^13^C-NMR spectra were taken in C_6_D_6_ or CDCl_3_ on a Bruker DPX-300 (300 MHz ^1^H-NMR and 75 MHz ^13^C-NMR) or a Bruker DRX-500 instrument (500 MHz ^1^H-NMR and 125 MHz ^13^C-NMR). The chemical shifts *(*δ) are reported in ppm using tetramethylsilane (TMS) as an internal standard. Data are reported as: s = singlet, d = doublet, t = triplet, q = quartet, sept = septuplet, br s = broad singlet, dd = doublet of doublets, dq = doublet of quartets, ddt = doublet of doublet of triplets, dddt = doublet of doublet of doublet of triplets, ddd = doublet of doublet of doublets, dddd = doublet of doublet of doublet of doublets, dtt = doublet of triplet of triplets, dtd = doublet of triplet of doublets, td = triplet of doublets, tt = triplet of triplets, qd = quartet of doublets, m = multiplet; coupling constant (s) in Hz; integration. *J*-resolved ^1^H-NMR experiments were run to extract chemical shifts *(*δ) and coupling constants (*J*) in crowded regions of the spectra. High resolution mass spectra (HRMS) were measured using ESI-Q-TOF in positive mode on micrOTOF II-ESI-TOF Mass Spectrometer-Bruker Daltonics. 

### 3.2. Experimental Procedures

*1-(3-Ethylisoxazol-5-yl)ethanol* (**2**): The preparation of isoxazolylalcohol **2** was described in reference [8]. IR ν_max_ (liquid film): 811, 899, 1079, 1107, 1144, 1426, 1463, 1601, 3377 cm^−1^. ^1^H-NMR, 500 MHz, (CDCl_3_), δ(ppm): 1.26 (t, 3H, *J* = 7.6 Hz); 1.56 (d, 3H, *J* = 6.7 Hz); 2.52 (br s, 1H); 2.67 (q, 2H, *J* = 7.6 Hz); 4.97 (q, 1H, *J* = 6.7 Hz); 6.06 (s, 1H). ^13^C {^1^H} NMR, 125 MHz, (CDCl_3_), δ(ppm): 12.6; 19.5; 21.8; 63.1; 99.4; 165.1; 175.9. HRESIMS: calcd for C_7_H_12_NO_2_^+^ (MH+) 142.0868; found 142.0862. 

*1-(3-Ethylisoxazol-5-yl)ethanone* (**3**): A solution of PCC (460 mg, 2.13 mmol) in CH_2_Cl_2_ (15 mL) was added to a solution of alcohol **2** (100 mg, 0.71 mmol) in CH_2_Cl_2_ (5 mL). After 6 h, the mixture was filtered through a sintered glass funnel with a silica gel and Celite^®^ bed, dried and washed several times with ethyl ether. The solvent of the filtrate was evaporated at reduced pressure and compound **3** was obtained with 90% yield (88.8 mg; 0.64 mmol). The crude product crystallized spontaneously at 0 °C. mp 31–32 °C. IR ν_max_ (liquid film): 610, 661, 849, 907, 963, 1090, 1188, 1295, 1368, 1470, 1581, 1698 cm^−1^. ^1^H-NMR, 500 MHz, (CDCl_3_), δ(ppm): 1.30 (t, 3H, *J =* 7.6 Hz); 2.60 (s, 3H); 2.77 (q, 2H, *J* = 7.6 Hz); 6.77 (s, 1H). ^13^C-NMR {^1^H}, 125 MHz, (CDCl_3_), δ(ppm): 12.5; 19.6; 27.2; 106.6; 166.0; 166.5; 187.1. 

*4-(1,3-Dioxan-2-yl)-2-(3-ethylisoxazol-5-yl)butan-2-ol* (**5**): Under an atmosphere of nitrogen, magnesium turnings (120 mg, 4.95 mmol) activated with iodine and THF (5 mL) were heated to reflux. Then, a solution of 2-(2-bromoethyl)-1,3-dioxane (967 mg, 4.95 mmol) in THF (5 mL) was added dropwise by addition funnel and the mixture was kept under reflux and magnetic stirring for about one hour, until the total consumption of magnesium. The temperature was lowered to 0 °C and a solution of compound **3** (230 mg, 1.65 mmol) in THF (5 mL) was added dropwise. The reaction mixture was kept stirring for 12 h at room temperature and further 6 h under reflux. Then, water (10 mL) was added and the mixture was extracted with diethyl ether (3 × 15 mL). Organic extracts were dried with anhydrous magnesium sulfate, filtered and the solvent removed at reduced pressure. The compound **5** was obtained with 95% yield (400.0 mg; 1.57 mmol) after purification by column chromatography on silica gel using hexane/ethyl acetate (60:40). IR ν_max_ (liquid film): 811, 896, 1004, 1145, 1241, 1287, 1378, 1418, 1464, 1597, 1858, 2942, 2973, 3417 cm^−1 1^H-NMR, 500 MHz, (C_6_D_6_), δ(ppm): 0.55 (dtt, 1H, *J* = 13.2; 11.2; 4.9 Hz); 1.01 (t, 3H, *J* = 7.6 Hz); 1.46 (s, 3H); 1.69 (dtt, 1H, *J* = 13.2; 3.1; 2.7 Hz); 1.77 (dtd, 1H, *J* = 13.3; 7.9; 4.7 Hz); 1.74 (dtd; 1H; *J* = 13.3; 7.9; 4.7 Hz); 2.01 (ddd, 1H, *J* = 14.5; 7.9; 6.8 Hz); 2.12 (ddd, 1H, *J* = 14.5; 7.9; 6.8 Hz); 2.40 (dq; 2H; *J* = 7.6; 15.5 Hz); 3.23 (ddd; 1H; *J* = 12.4; 11.2; 2.7 Hz); 3.20 (ddd; 1H; *J* = 12.4; 11.2; 2.7 Hz); 3.59 (br s, 3H) 3.71 (dddd; 1H; *J* = 12.4; 4.9; 3.1; 1.5 Hz); 3.69 (dddd; 1H; *J* = 12.4; 4.9; 3.1; 1.5 Hz); 4.22 (t, 1H, *J* = 4.7 Hz); 5.91 (s, 1H). ^13^C-NMR {^1^H}, 125 MHz, (C_6_D_6_), δ(ppm): 12.6; 19.8; 25.7; 28.2; 30.1; 35.8; 66.6; 71.6; 99.9; 101.9; 164.6; 178.2. HRESIMS: calcd for C_13_H_22_NO_4_^+^ (MH^+^) 256.1549; found 256.1543.

*(5Z)-6-Amino-1-(1,3-dioxan-2-yl)-3-hydroxy-3-methyloct-5-en-4-one* (**6**): A solution of compound **5** (100 mg; 0.39 mmol) in methanol (1.5 mL) was added to a suspension of 15% platinum on carbon (15 mg) in methanol (1.5 mL). The reaction mixture was kept under hydrogen at 7 atm for 72 h. The catalyst was filtered off and washed with ethyl acetate. Removal of the solvent under vacuum left a white solid with 91% yield (91.2 mg; 0.35 mmol). mp 93–94 °C. IR ν_max_ (liquid film): 640, 808, 814, 999, 1147, 1264, 1537, 1624, 2848, 2938, 2973, 3201, 3358, 3417 cm^−1 1^H-NMR, 500 MHz, (CDCl_3_), δ(ppm): 1.18 (t, 3H, *J* = 7.6 Hz); 1.30 (dtt, 1H, *J* = 13.2; 11.2; 4.9 Hz); 1.33 (s, 3H); 1.50 (m, 1H); 1.69 (m, 1H); 1.72 (dd, 2H, *J* = 6.9; 9.4 Hz); 2.05 (dtt, 1H, *J* = 13.2; 3.1; 2.7 Hz); 2.24 (dq, 2H, *J* = 7.6; 1.7 Hz); 3.76 (ddd, 1H, *J* = 12.4; 11.2; 2.7 Hz); 3.74 (ddd, 1H, *J* = 12.4; 11.2; 2.7 Hz); 4.10 (dddd, 1H, *J* = 12.4; 4.9; 3.1; 1.5 Hz); 4.08 (dddd, 1H, *J* = 12.4; 4.9; 3.1; 1.5 Hz); 4.50 (dd, 1H, *J* = 9.4; 6.0 Hz); 4.57 (br s, 1H); 5.14 (d, 1H, *J* = 1.7 Hz); 5.19 (br s, 1H); 9.59 (br s, 1H). ^13^C-NMR {^1^H}, 125 MHz, (CDCl_3_), δ(ppm): 12.0; 26.9; 29.6; 34.9; 66.8; 75.4; 88.2; 102.5; 168.7; 200.3. HRESIMS: calcd for C_13_H_24_NO_4_^+^ (MH^+^) 258.1705; found 258.1670.

*2-[2-(1,3-Dioxan-2-yl)ethyl]-5-ethyl-2-methylfuran-3(2H)-one* (**7**): A solution of **6** (100 mg; 0.39 mmol) in methanol (3 mL) was treated under stirring with aqueous hydrochloric acid (0.1 M; 0.50 mL). After one hour, a more concentrated solution of hydrochloric acid (1 M; 0.50 mL) was added and this treatment was repeated twice. The mixture was then neutralized with saturated solution of NaHCO_3_ and saturated with NaCl. The extraction with ethyl acetate (3 × 15 mL), drying with anhydrous magnesium sulfate and removal of the solvent gave **7** with 85% (79.6 mg; 0.33 mmol). IR ν_max_ (liquid film): 811, 926, 1007, 1145, 1241, 1287, 1383, 1450, 1591, 1703, 2853, 2927, 2972 cm^−1 1^H-NMR, 500 MHz, (CDCl_3_), δ(ppm): 1.15 (t, 3H, *J* = 7.5 Hz); 1.24 (dtt, 1H, *J* = 10.4; 4.9 Hz); 1.27 (s, 3H); 1.45 (dt, 2H, *J* = 8.4; 5.1 Hz); 1.77 (t, 2H, *J* = 8.4 Hz); 1.99 (dtt, 1H, *J* = 13.2; 4.9; 2.1 Hz); 2.42 (q, 2H, *J* = 7.5 Hz); 3.65 (ddd, 2H, *J* = 11.0; 10.4; 2.1 Hz); 4.01 (dt; 2H; *J* = 11.0; 4.9 Hz); 4.40 (t, 1H, *J* = 5.1 Hz); 5.30 (s, 1H). ^13^C-NMR {^1^H}, 125 MHz, (CDCl_3_), δ(ppm): 10.2; 21.8; 24.1; 25.7; 28.8; 30.8; 66.8; 90.3; 101.5; 101.6; 193.7; 206.9. HRESIMS: calcd for C_13_H_21_O_4_^+^ (MH^+^) 241.1440; found 241.1433.

*3-(5-Ethyl-2-methyl-3-oxo-2,3-dihydrofuran-2-yl)propanal* (**8**): Hydrochloric acid (2 M, 1.5 mL) was added to a solution of compound **7** (153 mg, 0.64 mmol) in methanol (3 mL) and ethyl acetate (1 mL). After 24 h, another portion of hydrochloric acid (2 M, 1.5 mL) was added. The mixture was kept stirring for 2 days at room temperature. Then, methanol was evaporated under reduced pressure, a saturated solution of NaHCO_3_ was added until complete neutralization of the acid and the mixture extracted with ethyl acetate (3 × 15 mL). The combined organic extracts were dried with anhydrous magnesium sulfate, filtered and the solvent removed at reduced pressure. The compound **8** was obtained in 60% (70 mg; 0.38 mmol) after purification by column chromatography on silica gel using hexane–ethyl acetate–methanol (60:30:10) as eluent. IR ν_max_ (liquid film): 811, 925, 1057, 1126, 1389, 1593, 1703, 2932, 2978 cm^−1 1^H-NMR, 500 MHz, (CDCl_3_), δ(ppm): 1.30 (t, 3H, *J* = 7.5 Hz); 1.38 (s, 3H); 2.11 (t, 2H, *J* = 6.0 Hz); 2.28 (qd; 2H; *J* = 7.5; 1.4 Hz); 2.38 (td, 2H, *J* = 6.0; 1.8 Hz); 5.43 (s, 1H); 9.71 (t, 1H, *J* = 1.8 Hz). ^13^C-NMR {^1^H}, 125 MHz, (CDCl_3_), δ(ppm): 10.2; 21.9; 24.1; 28.8; 37.8; 89.6; 101.8; 193.9; 200.6; 206.2. HRESIMS: calcd for C_10_H_15_O_3_^+^ (MH^+^) 183.1021; found 183.1016. 

*3-(5-Ethyl-2-methyl-3-oxo-2,3-dihydrofuran-2-yl)propanal* (**8**): Hydrochloric acid (0.1 M, 0.50 mL) was added to a solution of compound **6** (100 mg, 0.39 mmol) in methanol (3 mL). Keeping the mixture stirring at room temperature, additional portions of hydrochloric acid were added at one hour intervals (3 × 0.5 mL of 1 M HCl and then 4 × 0.5 mL of 2 M HCl). To end the reaction, a saturated solution of sodium bicarbonate was added until complete neutralization of the acid and the mixture extracted with ethyl acetate (3 × 15 mL). The combined organic extracts were dried with anhydrous magnesium sulfate, filtered and the solvent removed at reduced pressure. The compound **8** was obtained in 80% yield (85 mg; 0.47 mmol, 2 steps) after purification by column chromatography on silica gel using hexane/ethyl acetate/methanol (60:30:10) as eluent. IR ν_max_ (liquid film): 811, 925, 1057, 1126, 1389, 1593, 1703, 2932, 2978 cm^−1^ 1.30 (t, 3H, *J* = 7.5 Hz); 1.38 (s, 3H); 2.11 (t, 2H, *J* = 6.0 Hz); 2.28 (qd; 2H; *J* = 7.5; 1.4 Hz); 2.38 (td, 2H, *J* = 6.0; 1.8 Hz); 5.43 (s, 1H); 9.71 (t, 1H, *J* = 1.8 Hz). ^13^C-NMR {^1^H}, 125 MHz, (CDCl_3_), δ(ppm): 10.2; 21.9; 24.1; 28.8; 37.8; 89.6; 101.8; 193.9; 200.6; 206.2. HRESIMS: calcd for C_10_H_15_O_3_^+^ (MH^+^) 183.1021; found 183.1016. 

*5-Ethyl-2-(3-hydroxyhex-5-en-1-yl)-2-methylfuran-3(2H)-one* (**9**): *Preparation of the Grignard reagent:* A solution of allyl bromide (0.46 g, 0.33 mL, 3.8 mmol) in diethyl ether (2 mL) was added slowly to magnesium turnings (0.28 g, 11.4 mmol) activated with iodine and diethyl ether (1.2 mL), at 0 °C under an atmosphere of nitrogen, keeping the stirring for an additional hour. A portion of this solution (1.6 mL) was added dropwise, at room temperature, into a flask containing a solution of compound **8** (173.6 mg, 0.95 mmol) in THF (30 mL). Immediately there was formation of a white solid. After 1 h a saturated solution of ammonium chloride (15 mL) was added to dissolve the precipitate and the mixture was extracted with dichloromethane (4 × 15 mL) and ethyl acetate (4 × 15 mL). The combined organic extract was dried with anhydrous magnesium sulfate, filtered and the solvent removed at reduced pressure. The compound **9** in 52% yield (110.8 mg; 0.49 mmol) was obtained by column chromatography on silica gel using hexane/ethyl acetate/methanol (75:25:5) as eluent. IR ν_max_ (liquid film): 812, 923, 997, 1370, 1393, 1450, 1589, 1691, 2927, 2977, 3435 cm^−1 1^H-NMR, 500 MHz, (CDCl_3_), δ(ppm): 1.24 (t, 3H, *J* = 7.6 Hz); 1.25 (t, 3H, *J* = 7.6 Hz); 1.36 (s, 3H); 1.36 (m overlapped on the s, 1H); 1.50 (ddt, 1H, *J* = 14.3; 11.5; 4.9 Hz); 1.78 (ddd, 1H, *J* = 13.7; 11.5; 4.9 Hz); 1.95 (ddd, 1H, *J* = 13.7; 11.5; 4.9 Hz); 2.12 (dtt, 1H, *J* = 14.0; 10.4; 1.8 Hz); 2.26 (dddt, 1H, *J* = 14.5; 6.8; 5.2; 1.8 Hz); 2.51 (qd, 2H, *J* = 7.6; 1.8 Hz); 3.59 (tt, 1H, *J* = 7.5; 4.5 Hz); 5.11 (ddt, 1H, *J* = 17.0; 2.5; 1.8 Hz); 5.14 (ddt, 1H, *J* = 11.2; 2.5; 1.8 Hz); 5.40 (s, 1H); 5.78 (dddd, 1H, *J* = 17.0; 11.2; 10.4; 6.8 Hz). ^13^C-NMR {^1^H}, 125 MHz, (CDCl_3_), δ(ppm): 10.3; 22.0 (21.8); 24.2; 30.0; 32.6 (32.5); 41.9 (41.7); 70.2 (70.4); 90.7; 101.6 (101.6); 118.3; 134.4; 193.9 (193.7); 207.3 (207.2). The number in parenthesis refers to duplicated signal assigned to the other diastereoisomer. HRESIMS: calcd for C_13_H_21_O_3_^+^ (MH^+^) 225.1491; found 225.1484.

*2-(3-Hydroxyhex-5-en-1-yl)-5-isopropyl-2-methylfuran-3(2H)-one* (**10**): Under nitrogen atmosphere and 0 °C, sodium hydride (31.8 mg, 79.4 mmol, 60% in mineral oil) was dissolved in dimethylformamide (2 mL). Then, the compound **9** (137 mg, 0.61 mmol) in dimethylformamide (7 mL) was added and maintained for 30 minutes under these conditions. Then methyl iodide was added (0.43 g, 0.19 mL, 3.05 mmol) at 0 °C and the reaction was maintained under these conditions for 3 h and then another 1 h at room temperature. The compound **10** was obtained after column chromatographic purification on silica gel using hexane–ethyl acetate–methanol (75:25:5) as eluent. IR ν_max_ (liquid film): 806, 1070, 1287, 1585, 1699, 2877, 2932, 2976, 3432 cm^−1 1^H-NMR, 500 MHz, (CDCl_3_), δ(ppm): 1.26 (2 d overlapped, 6H, *J* = 9.0 Hz); 1.36 (s, 3H); 1.36 (m overlapped on the s, 1H); 1.47 (ddt, 1H, *J* = 14.3; 12.5; 5.9 Hz); 1.78 (ddd, 1H, *J* = 14.5; 12.5 e 5.9 Hz); 1.96 (ddd, 1H, *J* = 14.5; 13.0; 5.9 Hz); 2.02 (br s, 1H); 2.11 (dtt, 1H, *J* = 14.5; 9.3; 2.0 Hz); 2.25 (dddt, 1H, *J* = 14.5; 7.7; 5.9; 2.0 Hz); 2,74 (sept, 1H, *J* = 9.0 Hz); 3.59 (tt, 1H, *J* = 9.3; 5.9 Hz); 5.11 (ddt, 1H, *J* = 17.2; 2.5; 2.0 Hz); 5.13 (ddt, 1H, *J* = 11.6; 2.5; 2.0 Hz); 5.38 (s, 1H); 5.80 (dddd, 1H, *J* = 17.2; 11.6; 9.3; 7.7 Hz). ^13^C-NMR {^1^H}, 125 MHz, (CDCl3), δ(ppm): (19.6) 19.5; 22.1 (21.9); 27.4; 30.0; 30.3; 32.6 (32.5); 41.9 (41.7); (70.4) 70.1; 90.5; 100.3 (101.6); 118.3 (118.2); 134.4; 197.5 (197.3); 207.5 (207.3). The number in parenthesis refers to duplicated signal assigned to the other diastereoisomers. HRESIMS: calcd for C_14_H_23_O_3_^+^ (MH^+^) 239.3306; found: 239.1642. 

*2-(3-Hydroxyhex-5-en-1-yl)-5-isopropenyl-2-methylfuran-3(2H)-one* (**11**): Under nitrogen atmosphere, *n*-butyllithium (1.39 M, 2.1 mL, 3 mmol) was added to a solution of diisopropylamine (0.30 g, 0.42 mL, 3 mmol) in THF (0.92 mL) at 0 °C. After stirring for 5 minutes under these conditions, the temperature of the reaction mixture was lowered to −78 °C and a solution of compound **9** in THF (2.5 mL) and HMPA (0.45 mL) was added (66.6 mg, 0.3 mmol). The mixture was kept under stirring for 45 minutes at −78 °C and dimethylmethyleneammonium iodide (1.2 g, 6.5 mmol) was added rapidly to the mixture, keeping the stirring for 5 h at −78 °C. Then the temperature was elevated to room temperature and the solvent removed at reduced pressure. Methanol (4.6 mL) and methyl iodide (3.5 mL) were added and the mixture was kept under stirring for 36 h at room temperature. Then the solvent was removed under reduced pressure and to the residue was added a 5% NaHCO_3_ aqueous solution (14.6 mL) and CH_2_Cl_2_ (11.6 mL). The mixture was kept stirring at room temperature for 4 h. Then the reaction mixture was acidified with hydrochloric acid (3 M, 10 mL) and stirred for 15 minutes at room temperature. Finally, the phases were separated and the aqueous phase was extracted with dichloromethane (4 × 15 mL) and ethyl acetate (4 × 15 mL). The combined organic extract was dried with anhydrous magnesium sulfate, filtered and the solvent removed at reduced pressure. The compound **10** in 48% yield (34 mg; 0.14 mmol; 4 steps) was obtained after purification by column chromatography on silica gel using hexane–ethyl acetate–methanol (70:25:5) as eluent. IR ν_max_ (liquid film): 812, 924, 1369, 1448, 1558, 1635, 1686, 2868, 2930, 2977, 3437 cm^−1 1^H-NMR, 500 MHz, (CDCl_3_), δ(ppm): 1.40 (s, 3H); 1.40 (m overlapped on the s, 1H); 1.51 (ddt, 1H, *J* = 14.5; 11.2; 4.9 Hz); 1.91 (ddd, 1H, *J* = 13.5; 11.2; 4.9 Hz); 1.98 (ddd, 1H, *J* = 13.5; 11.4; 4.9 Hz); 2.02 (dd, 3H, *J* = 1.7; 0.9 Hz); 2.10 (dtt, 1H, *J* = 14.5; 9.0; 2.0 Hz); 2.26 (dddt, 1H, *J* = 14.5; 6.2; 4.9; 2.0 Hz); 3.59 (tt, 1H, *J* = 9.0; 4.9 Hz); 5.11 (ddt, 1H, *J* = 15.7; 2.5; 2.0 Hz); 5.14 (ddt, 1H, *J* = 10.0; 2.5; 2.0 Hz); 5.44 (m, 1H); 5.59 (s, 1H); 5.78 (dddd, 1H, *J* = 15.7; 10.0; 9.0; 6.2 Hz). ^13^C-NMR {^1^H}, 125 MHz, (CDCl_3_), δ(ppm): 18.9; 22.1 (21.9); 30.1; 33.0 (32.9); 41.8 (41.7); 70.2 (70.4); 90.6; 101.3 (101.6); 118.3; 121.5 (121.4); 133.6; 134.5; 184.1 (184.0); 207.4 (207.2). The number in parenthesis refers to duplicated signal assigned to the other diastereoisomer. HRESIMS: calcd for C_14_H_21_O_3_^+^ (MH^+^) 237.1491; found 237.1485. 

## 4. Conclusions 

We have prepared a short series of 3(2*H*)-furanones through a very efficient method that uses an isoxazole as intermediate. An initial functionalized side chain introduced in the isoxazole can be modified later, leading easily to a large number of compounds having a common core structure, the furanone ring, that makes the method suitable for the preparation of libraries of compounds for biological activity studies. The presence of double bonds and/or functional groups in the side-chains also makes the compounds useful as starting materials for the synthesis of natural products.

## Supplementary Materials

Supplementary materials can be accessed at: http://www.mdpi.com/1420-3049/17/10/12151/s1.
