# Supplementary material for: Side-chain Modifications of Highly Functionalized 3(2H)-Furanones
Source: Molecules. 2012 Oct 16;17(10):12151–62. doi: 10.3390/molecules171012151 (PMC6268025; doi:10.3390/molecules171012151)

Supplementary Materials

Figure S1. ^{1}H-NMR spectrum of compound **2** (500 MHz, $CDCl_3$).

Acquisition Time (sec)	3.8535	***Comment***	Imported from UXNMR.			***Date***			
File Name				***Frequency (MHz)***	500.13	***Nucleus***	1H	***Number of Transients***	16
Original Points Count	32768	***Points Count***	32768	***Pulse Sequence***	zg30	***Solvent***	CHLOROFORM-D		
Sweep Width (Hz)	8503.40	***Temperature (degree C)***	27.000						

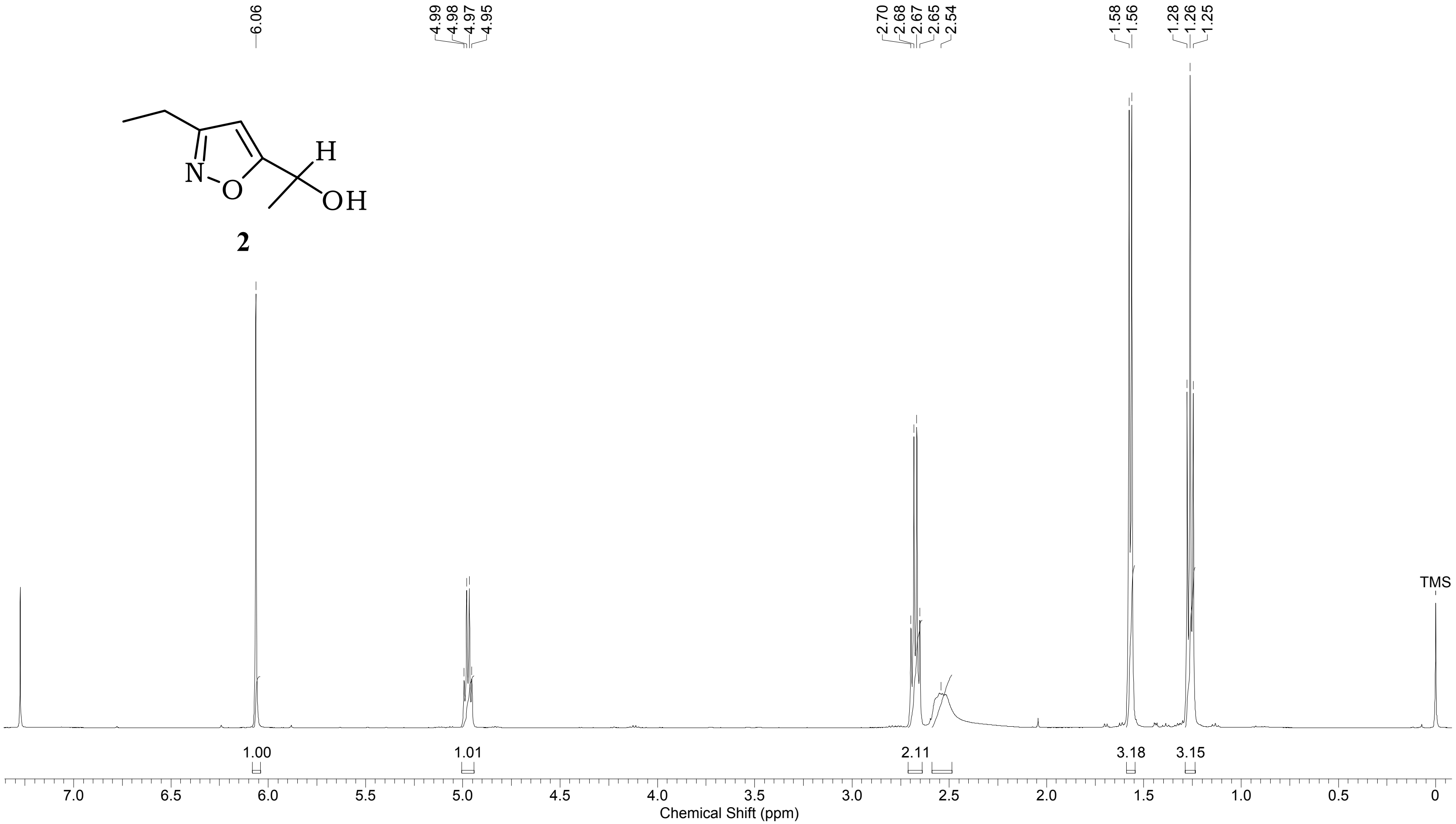

Figure S2. ^{13}C-NMR spectrum of compound **2** (125 MHz, $CDCl_3$).

Acquisition Time (sec)	1.0420	***Comment***	Imported from UXNMR.	***Date***			
File Name		***Frequency (MHz)***	125.76	***Nucleus***	13C	***Number of Transients***	3072
Original Points Count	32768	***Points Count***	32768	***Pulse Sequence***	zgpg30	***Solvent***	CHLOROFORM-D
Sweep Width (Hz)	31446.54	***Temperature (degree C)***	27.000				

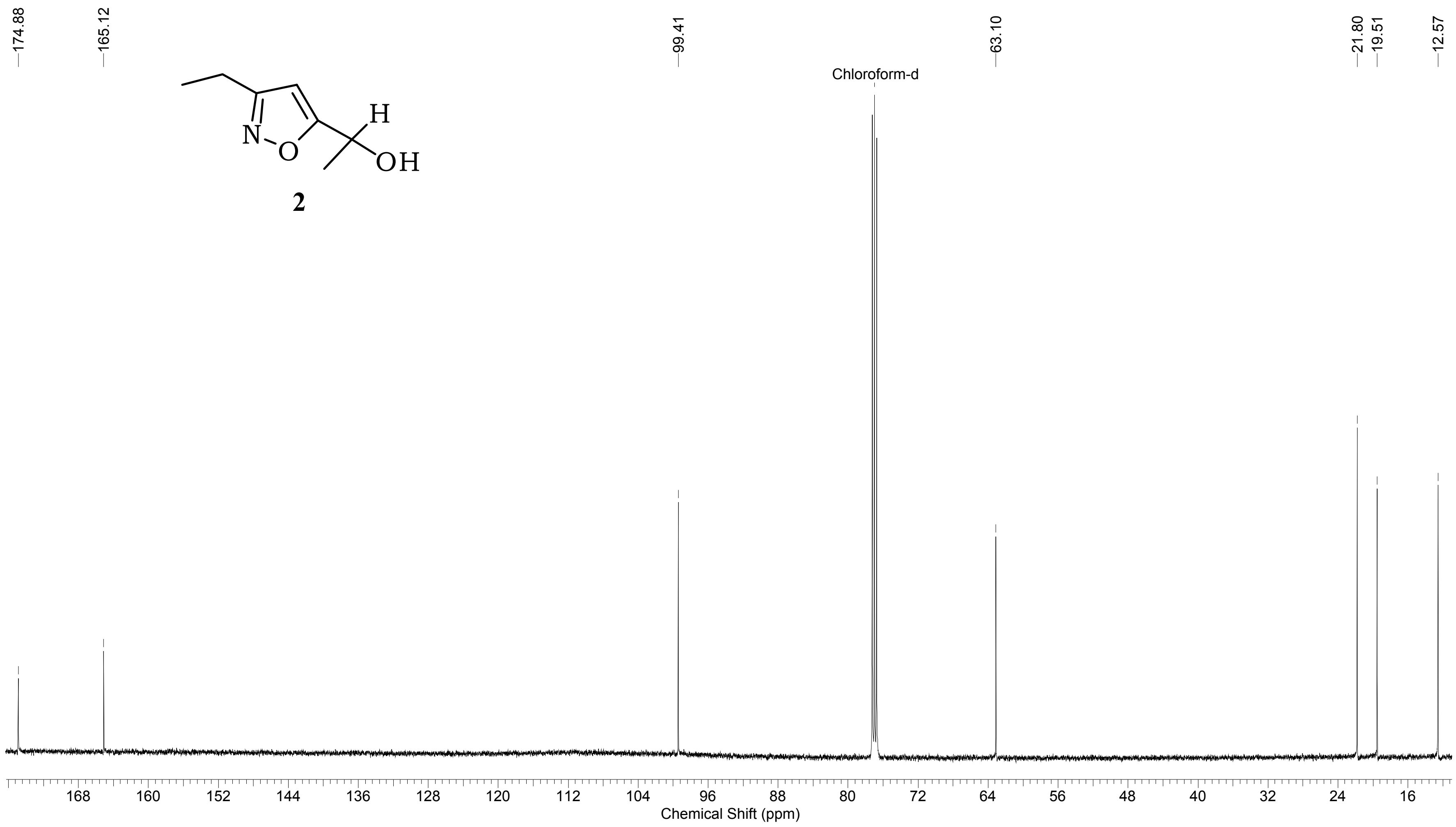

Figure S3. ^{13}C-NMR (DEPT 135) spectrum of compound **2** (125 MHz, $CDCl_3$).

Acquisition Time (sec)	1.0420	***Comment***	Imported from UXNMR.			***Date***	
File Name				***Frequency (MHz)***	125.76	***Nucleus***	13C
Number of Transients	1536						
Original Points Count	32768	***Points Count***	32768	***Pulse Sequence***	dept135	***Solvent***	CHLOROFORM-D
Sweep Width (Hz)	31446.54	***Temperature (degree C)***	27.000				

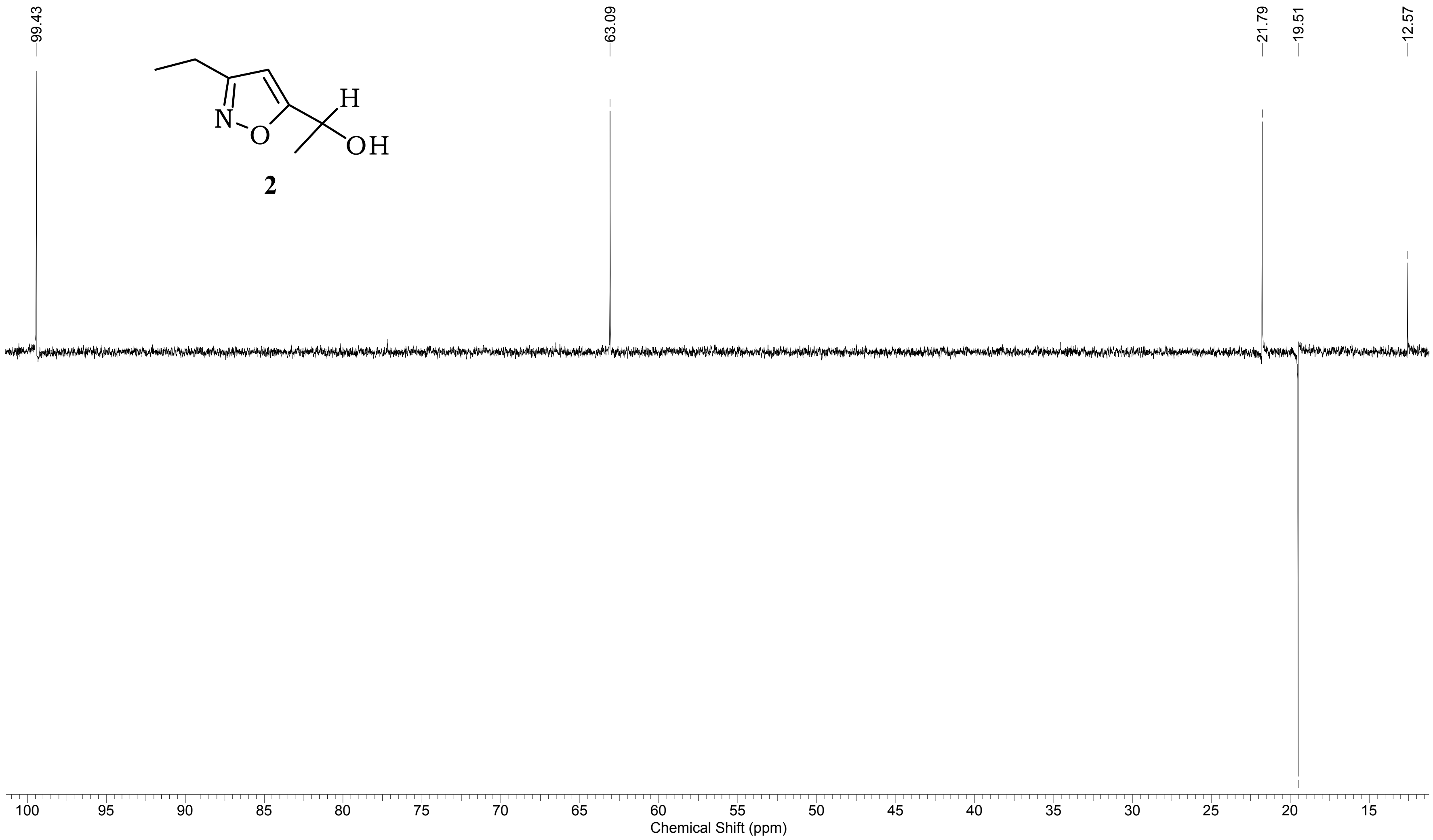

Figure S4. ^{1}H-NMR spectrum of compound **3** (500 MHz, $CDCl_3$).

Acquisition Time (sec)	3.8535	***Comment***	Imported from UXNMR.			***Date***			
File Name				***Frequency (MHz)***	500.13	***Nucleus***	1H	***Number of Transients***	16
Original Points Count	32768	***Points Count***	32768	***Pulse Sequence***	zg30	***Solvent***	CHLOROFORM-D		
Sweep Width (Hz)	8503.40	***Temperature (degree C)***	27.000						

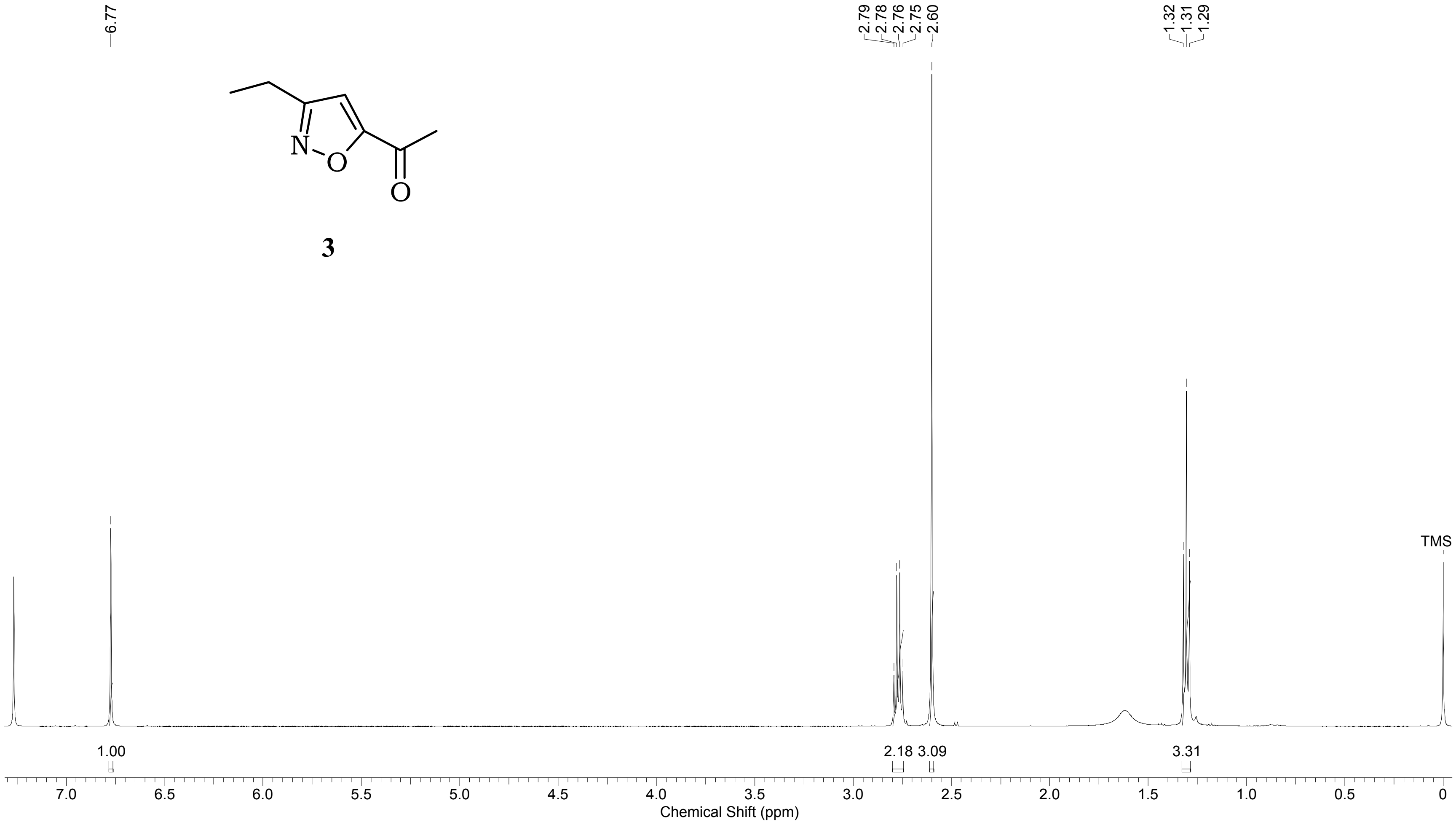

Figure S5. ^{13}C-NMR spectrum of compound **3** (125 MHz, $CDCl_3$).

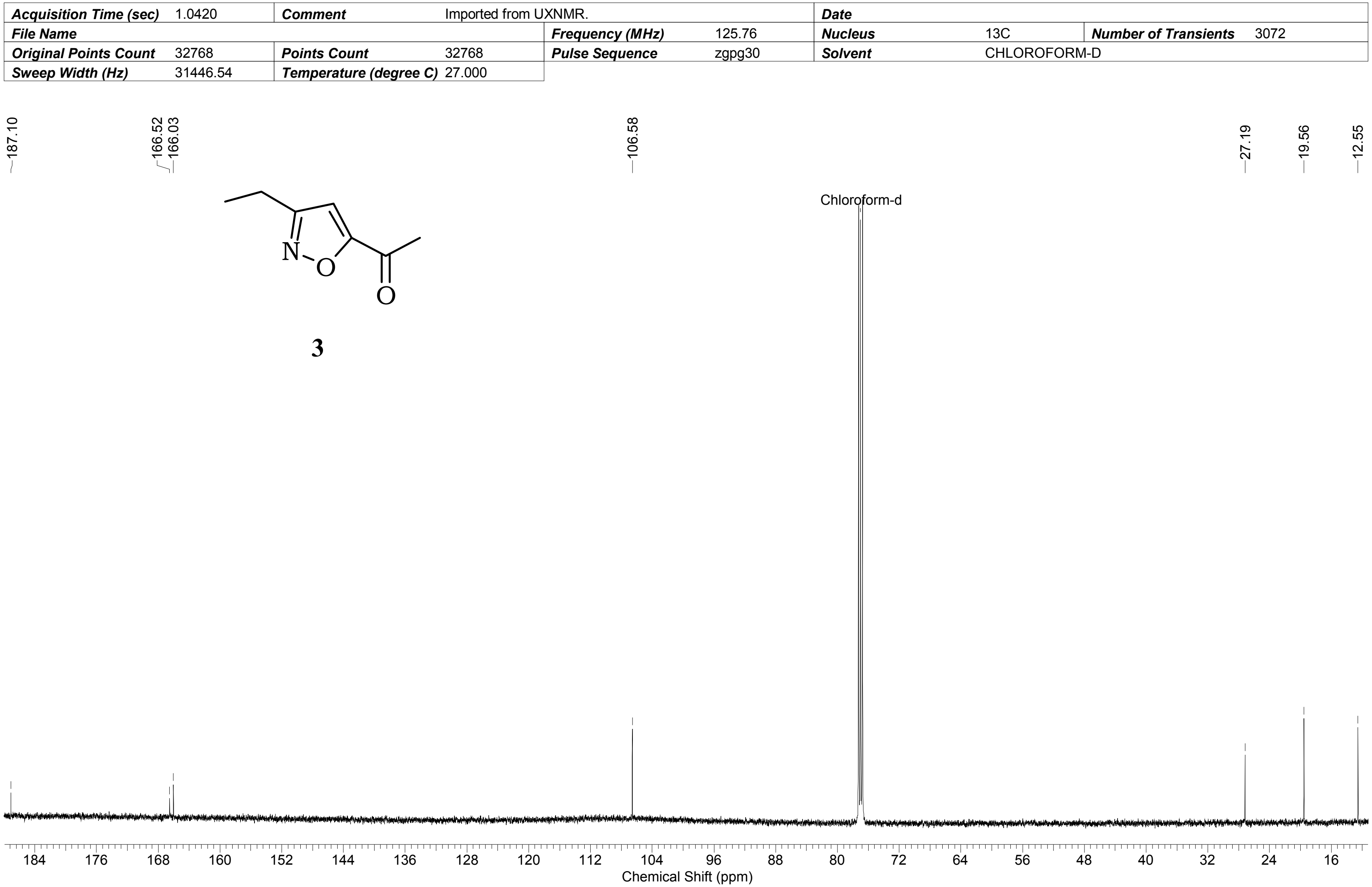

Figure S6. ^{13}C-NMR (DEPT 135) spectrum of compound **3** (125 MHz, $CDCl_3$).

Acquisition Time (sec)	1.0420	***Comment***	Imported from UXNMR.			***Date***			
File Name				***Frequency (MHz)***	125.76	***Nucleus***	13C	***Number of Transients***	1536
Original Points Count	32768	***Points Count***	32768	***Pulse Sequence***	dept135	***Solvent***	CHLOROFORM-D		
Sweep Width (Hz)	31446.54	***Temperature (degree C)***	27.000						

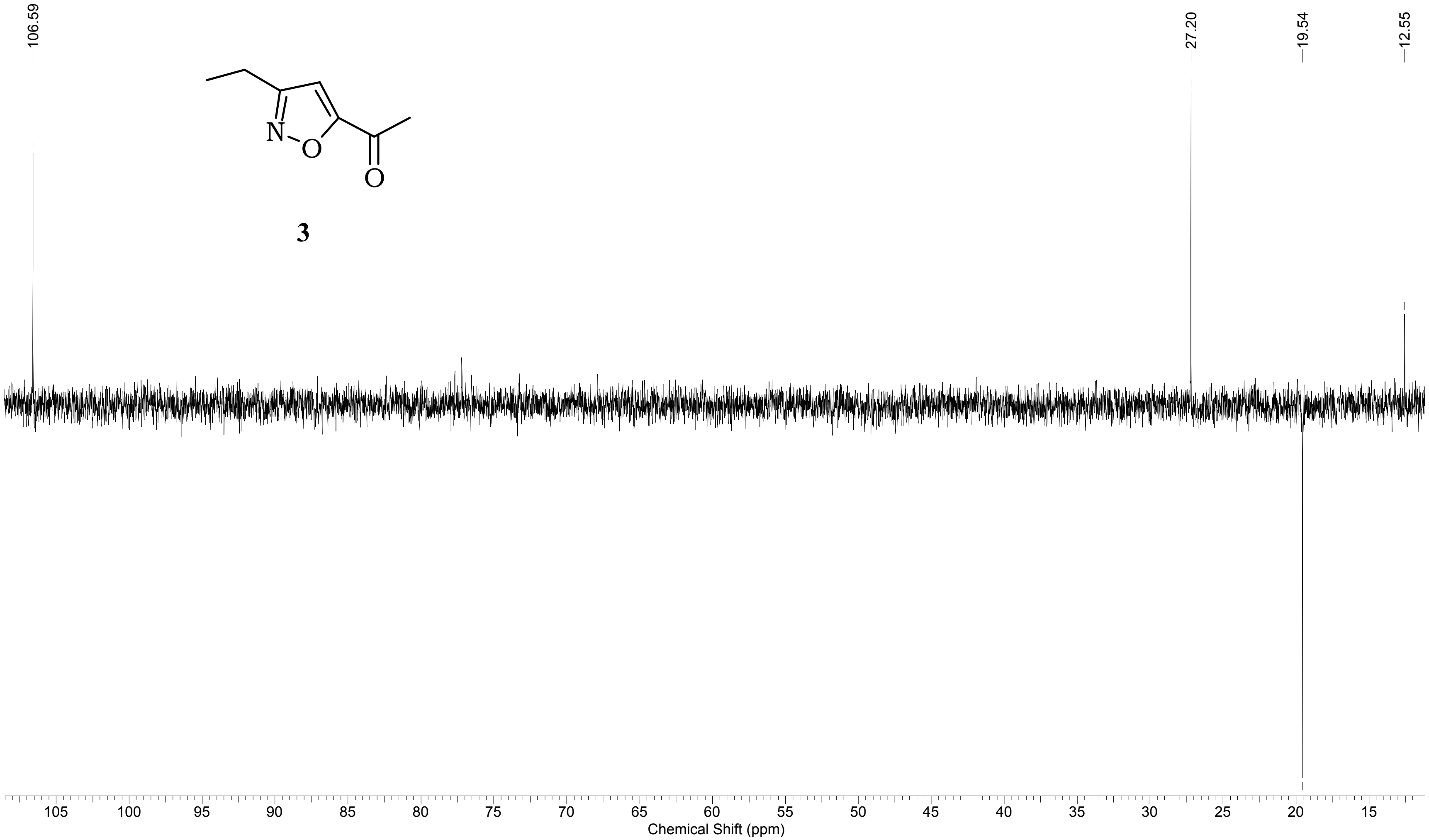

Figure S7. ^{1}H-NMR spectrum of compound **5** (500 MHz, C_6D_6).

Acquisition Time (sec)	3.8535	***Comment***	Imported from UXNMR.			***Date***	
File Name				***Frequency (MHz)***	500.13	***Nucleus***	1H
Original Points Count	32768	***Points Count***	32768	***Pulse Sequence***	zg30	***Solvent***	BENZENE-D6
Sweep Width (Hz)	8503.40	***Temperature (degree C)***	27.000				

Number of Transients 16

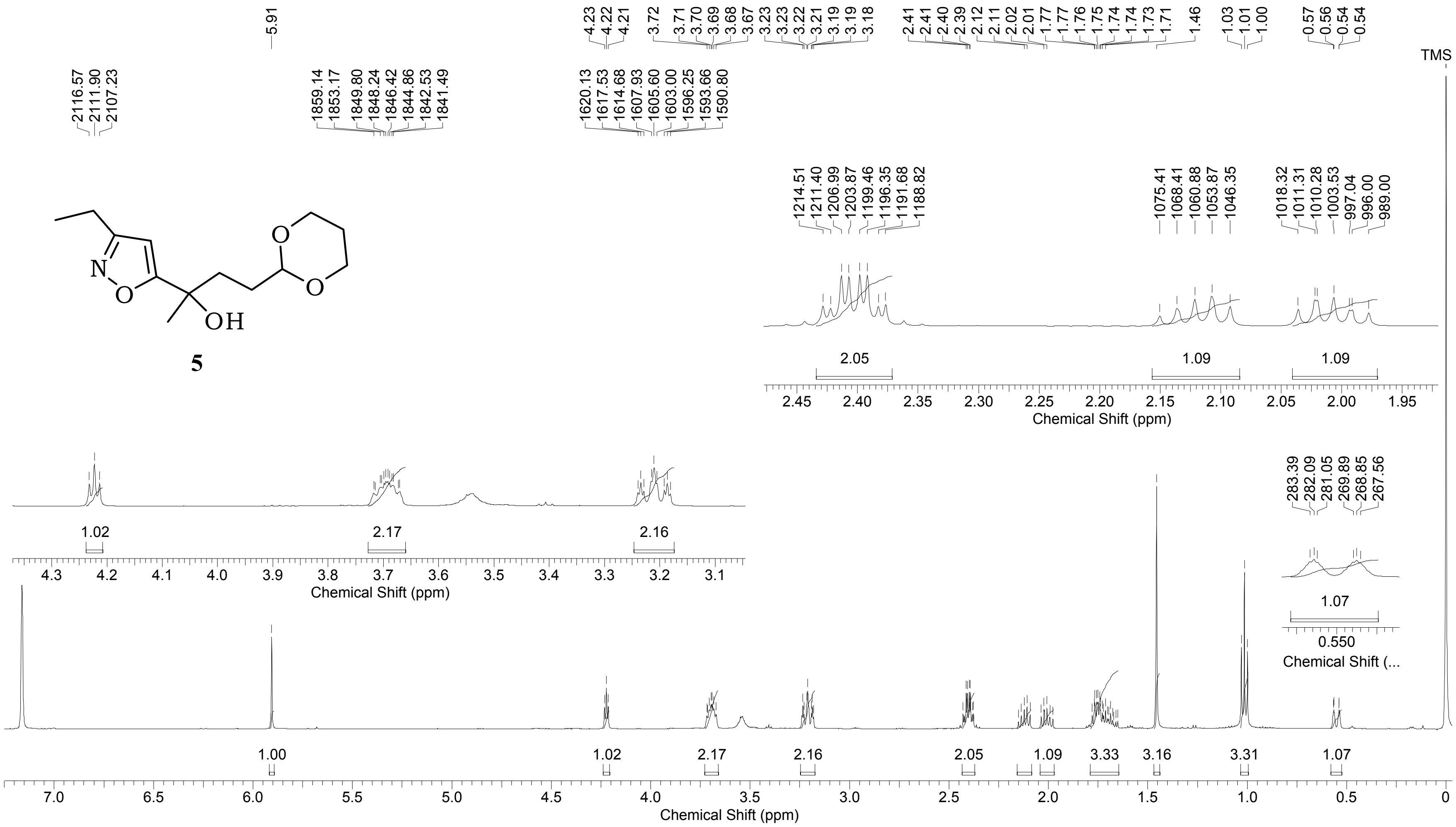

Figure S8. ^{13}C-NMR spectrum of compound **5** (125 MHz, C_6D_6).

Acquisition Time (sec)	1.0420	***Comment***	Imported from UXNMR.			***Date***	
File Name				***Frequency (MHz)***	125.76	***Nucleus***	13C
Number of Transients	3072						
Original Points Count	32768	***Points Count***	32768	***Pulse Sequence***	zgpg30	***Solvent***	CHLOROFORM-D
Sweep Width (Hz)	31446.54	***Temperature (degree C)***	27.000				

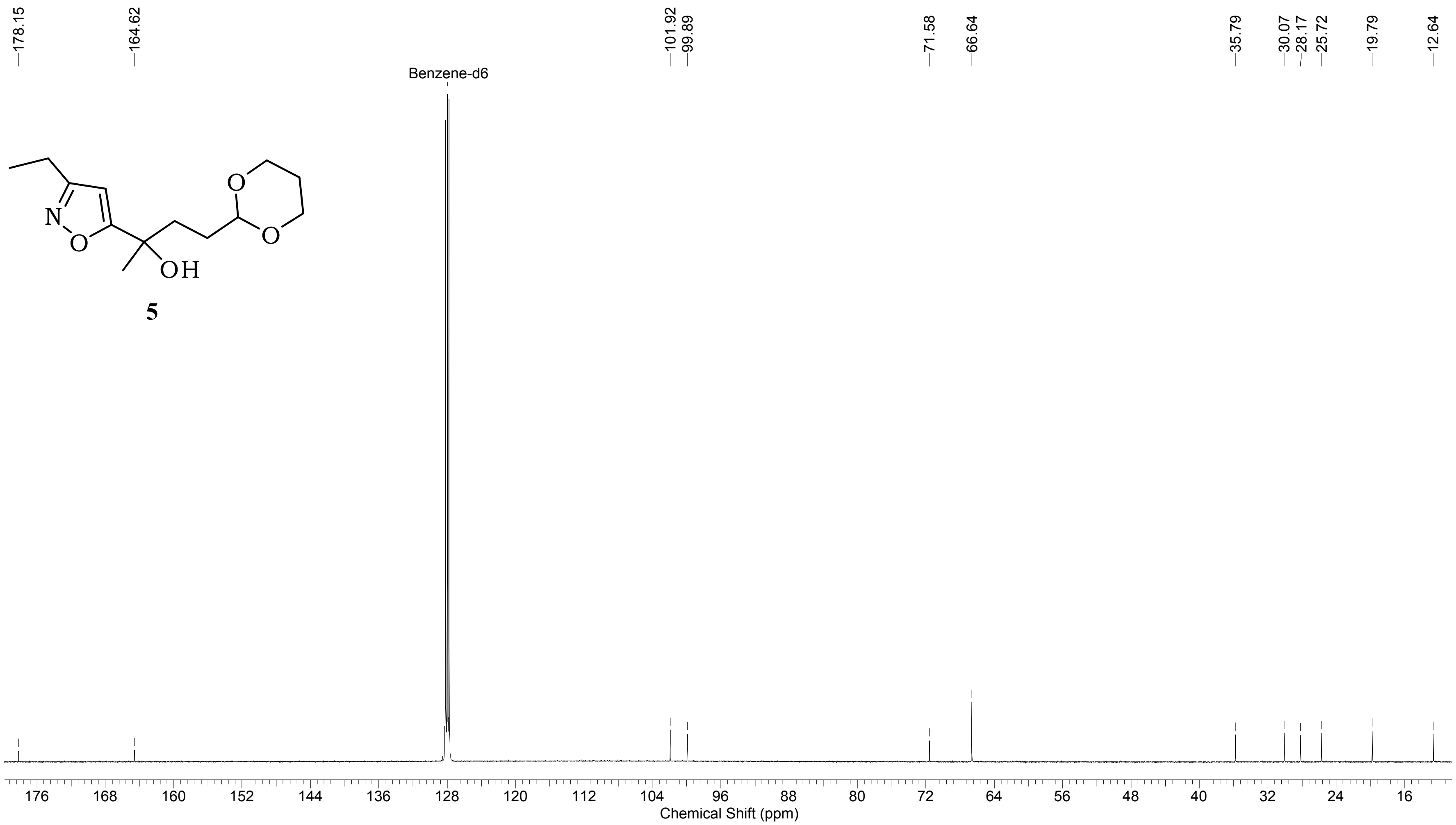

Figure S9. ^{13}C-NMR (DEPT 135) spectrum of compound **5** (125 MHz, C_6D_6).

Acquisition Time (sec)	1.0420	***Comment***	Imported from UXNMR.			***Date***			
File Name				***Frequency (MHz)***	125.76	***Nucleus***	13C	***Number of Transients***	1536
Original Points Count	32768	***Points Count***	32768	***Pulse Sequence***	dept135	***Solvent***	CHLOROFORM-D		
Sweep Width (Hz)	31446.54	***Temperature (degree C)***	27.000						

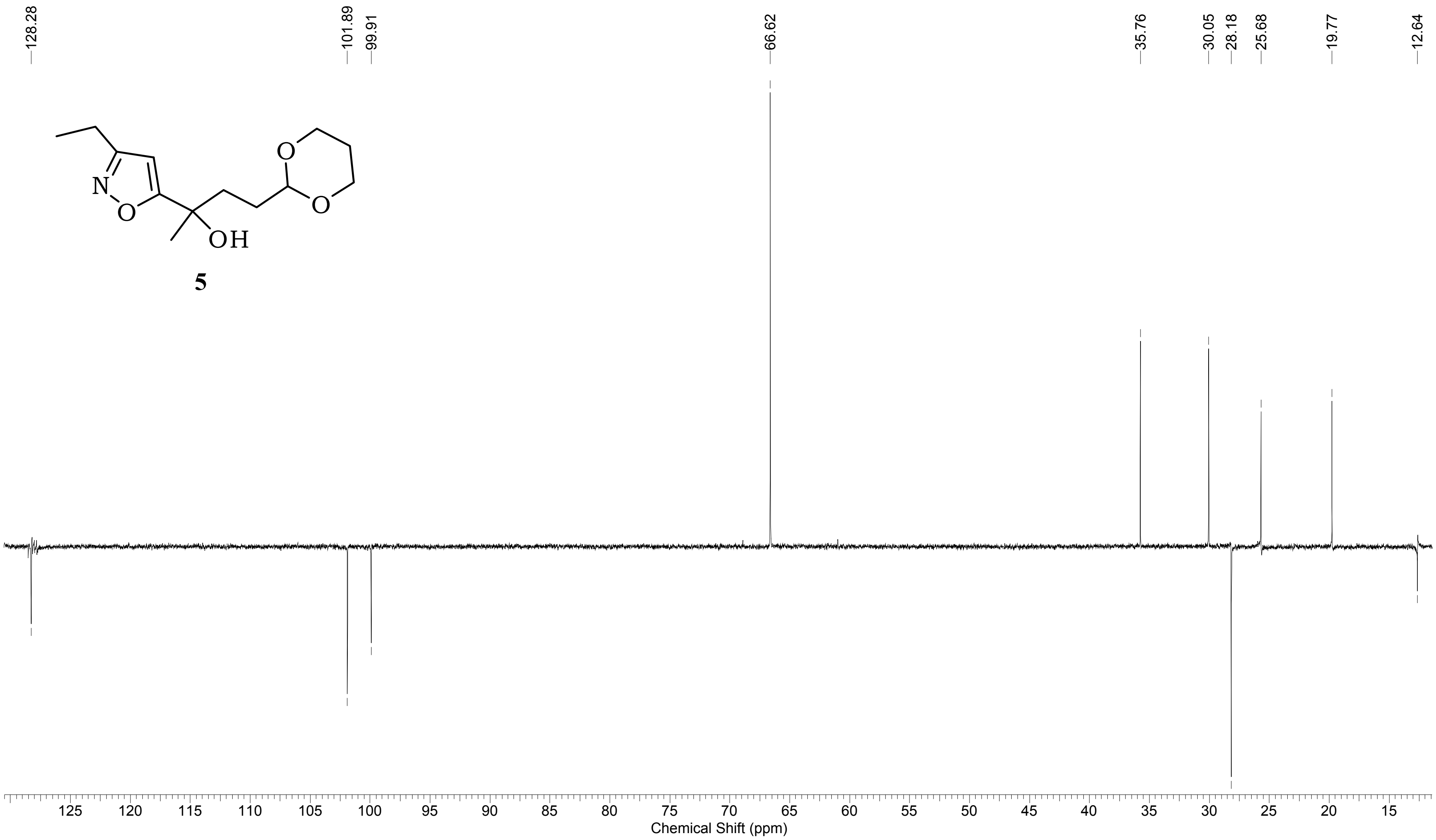

Figure S10. ^{1}H-NMR spectrum of compound **6** (500 MHz, $CDCl_3$).

Acquisition Time (sec)	7.7070	***Comment***	Imported from UXNMR.		***Date***		
File Name				***Frequency (MHz)*** 500.13	***Nucleus***	1H	***Number of Transients*** 16
Original Points Count	65536	***Points Count***	32768	***Pulse Sequence*** zg30	***Solvent***	CHLOROFORM-D	
Sweep Width (Hz)	8503.40	***Temperature (degree C)***	27.000				

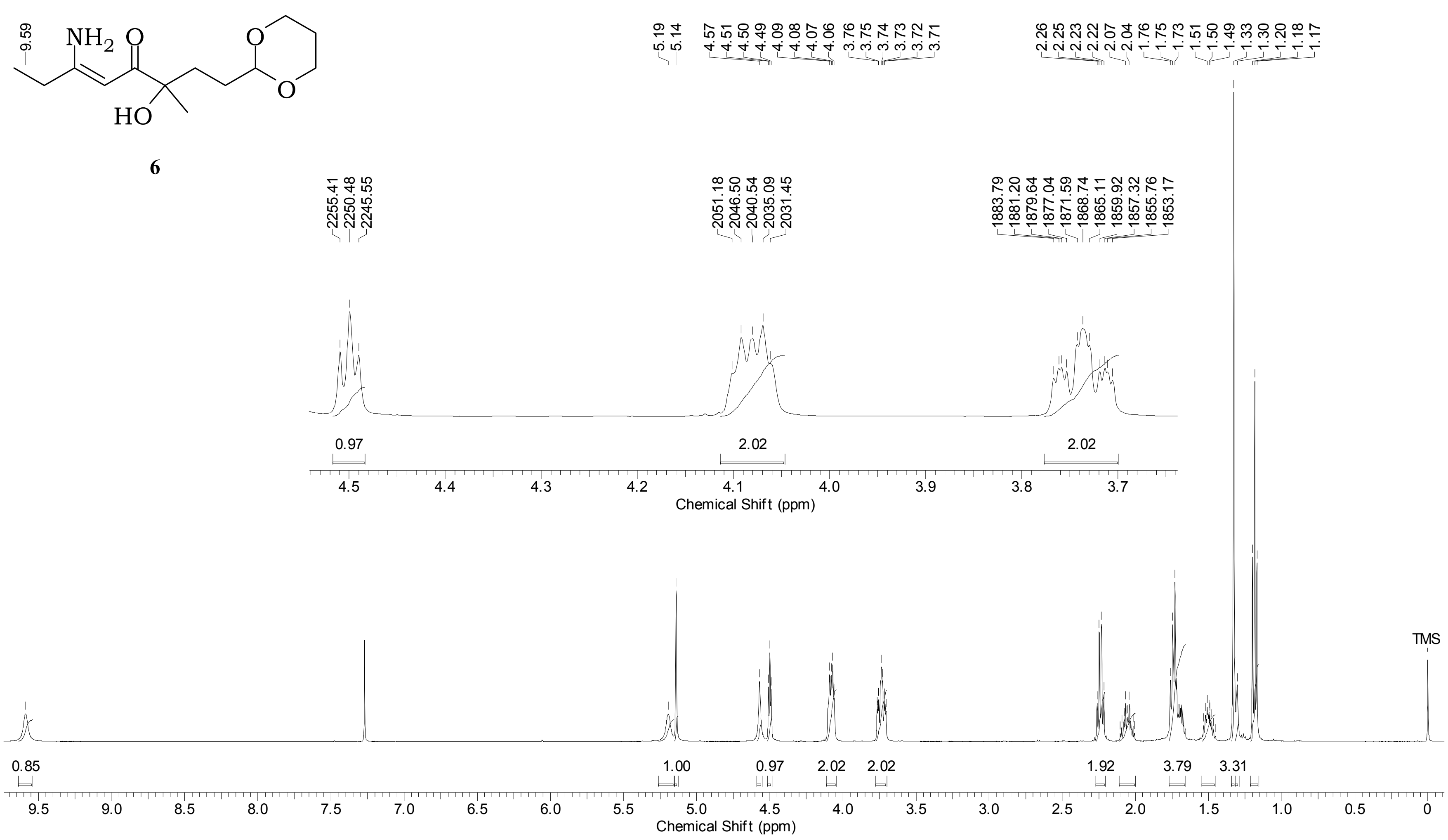

Figure S11. ^{13}C-NMR spectrum of compound **6** (125 MHz, $CDCl_3$).

Acquisition Time (sec)	1.0420	***Comment***	Imported from UXNMR.			***Date***			
File Name				***Frequency (MHz)***	125.76	***Nucleus***	13C	***Number of Transients***	1024
Original Points Count	32768	***Points Count***	32768	***Pulse Sequence***	zgpg30	***Solvent***	CHLOROFORM-D		
Sweep Width (Hz)	31446.54	***Temperature (degree C)***	27.000						

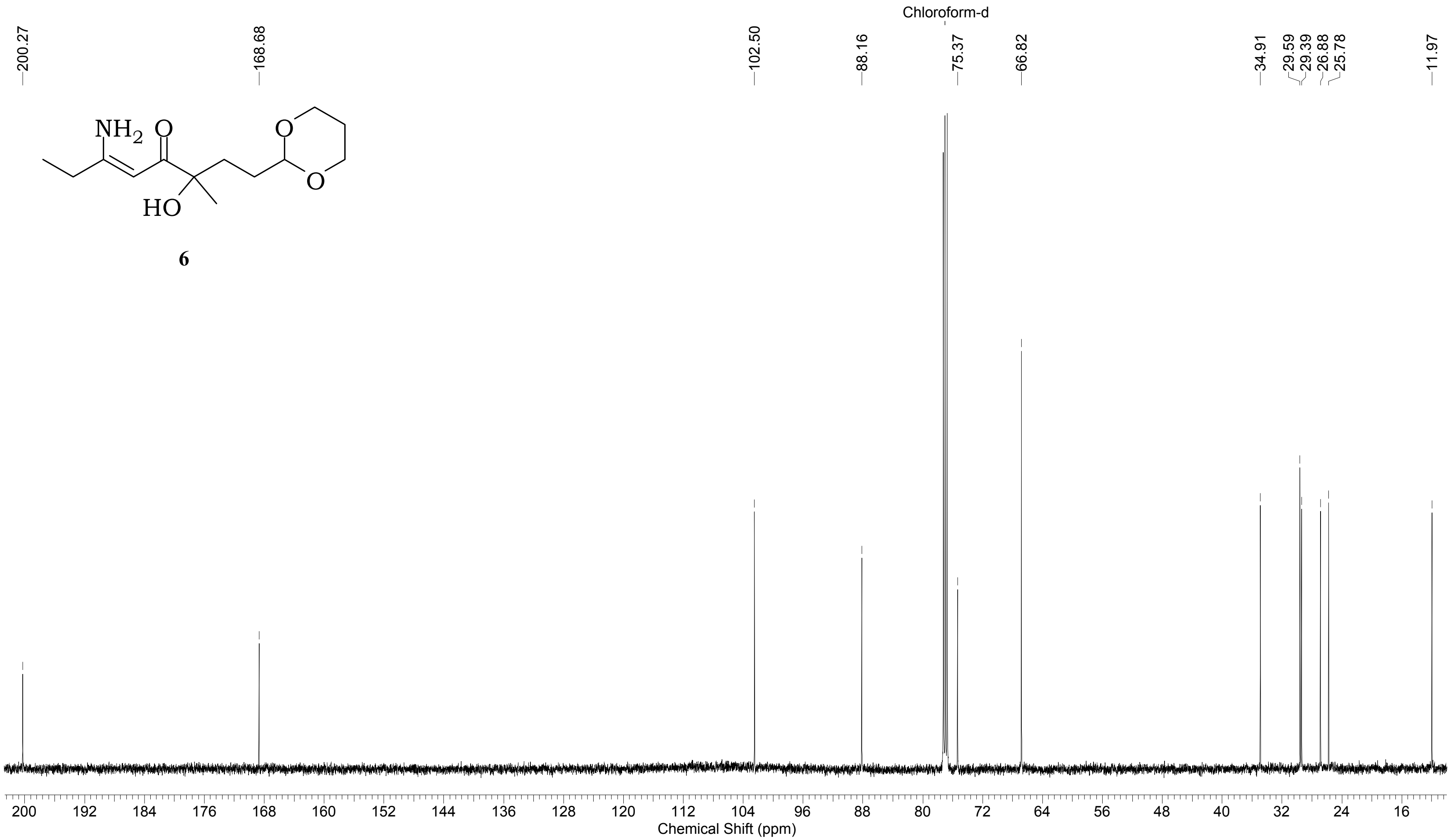

Figure S12. ^{13}C-NMR (DEPT 135) spectrum of compound **6** (125 MHz, $CDCl_3$).

Acquisition Time (sec)	1.0420	***Comment***	Imported from UXNMR.			***Date***			
File Name				***Frequency (MHz)***	125.76	***Nucleus***	13C	***Number of Transients***	512
Original Points Count	32768	***Points Count***	32768	***Pulse Sequence***	dept135	***Solvent***	CHLOROFORM-D		
Sweep Width (Hz)	31446.54	***Temperature (degree C)***	27.000						

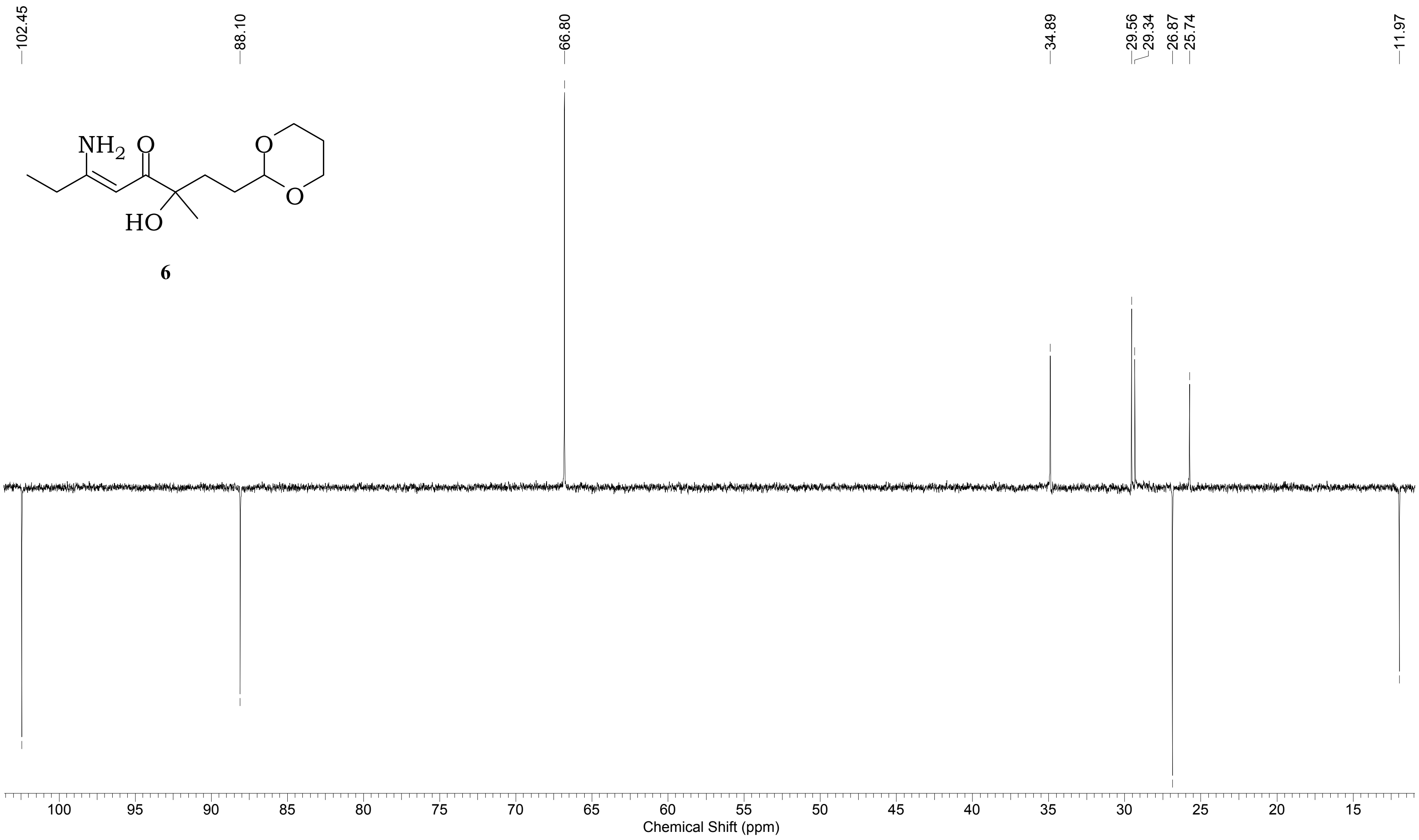

Figure S13. NOE spectrum of compound **6** (125 MHz, $CDCl_3$).

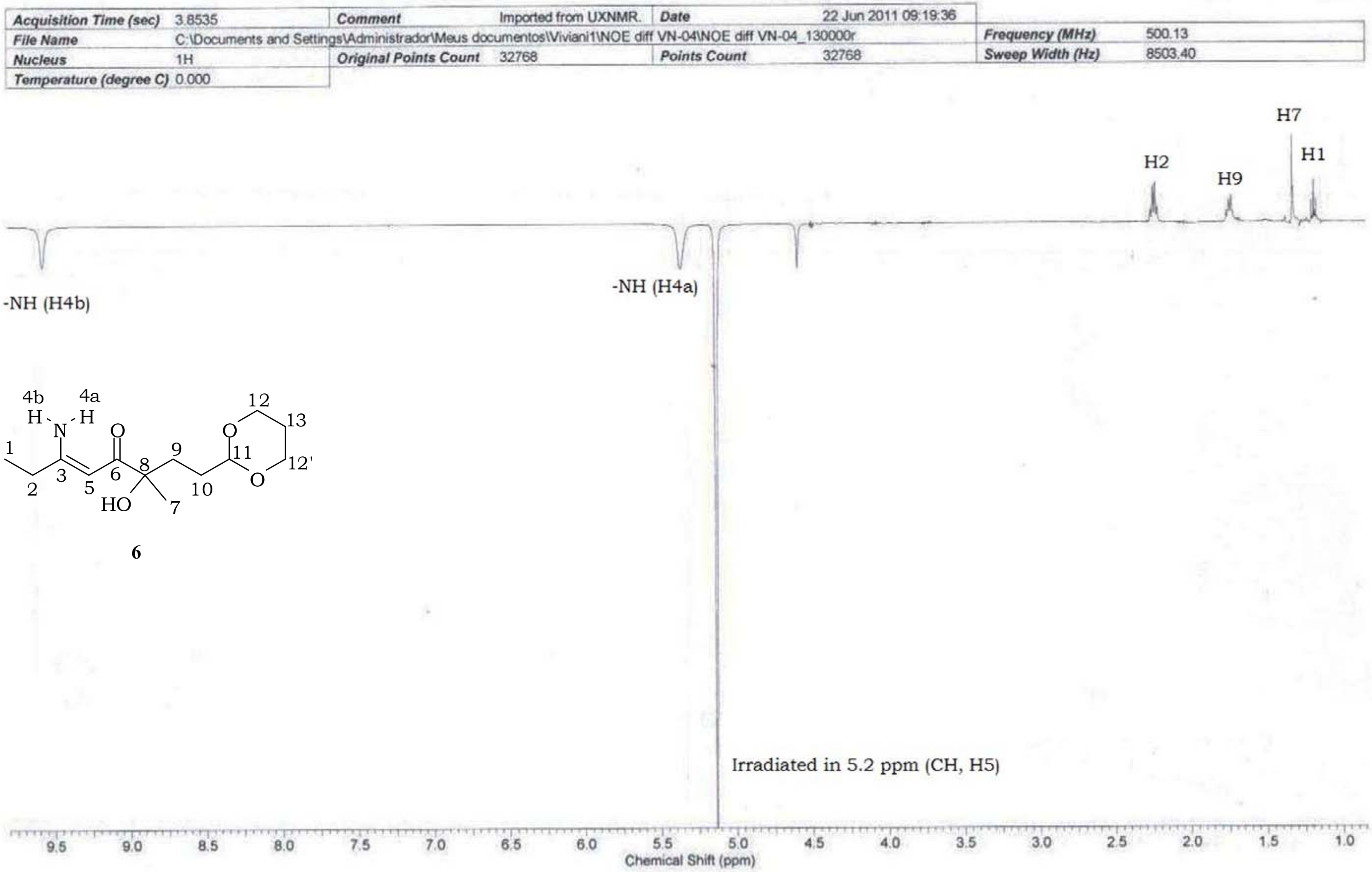

Figure S14. ^{1}H-NMR spectrum of compound **7** (500 MHz, $CDCl_3$).

Acquisition Time (sec)	7.7070	***Comment***	Imported from UXNMR.	***Date***	
File Name				***Frequency (MHz)***	500.13
Nucleus	1H	***Number of Transients***	16	***Original Points Count*** 65536	***Points Count*** 32768
Pulse Sequence	zg30	***Solvent***	CHLOROFORM-D	***Sweep Width (Hz)***	8503.40
Temperature (degree C)	27.000				

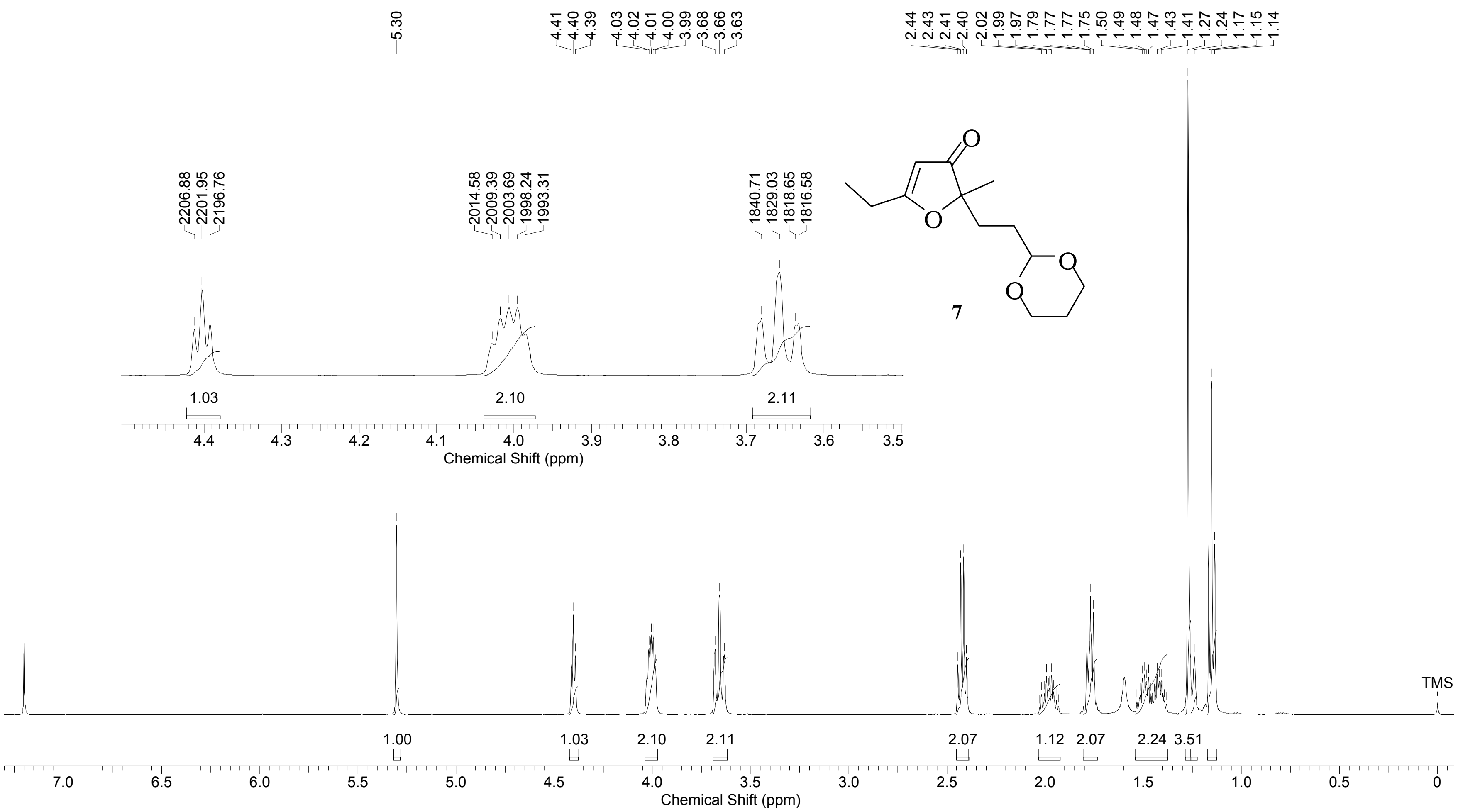

Figure S15. ^{13}C-NMR spectrum of compound **7** (125 MHz, $CDCl_3$).

Acquisition Time (sec) 1.0420	***Comment***	Imported from UXNMR.		***Date***	
File Name		***Frequency (MHz)***	125.76	***Nucleus***	13C
Number of Transients 1024	***Original Points Count*** 32768	***Points Count***	32768	***Pulse Sequence***	zgpg30
Solvent CHLOROFORM-D		***Sweep Width (Hz)***	31446.54	***Temperature (degree C)***	27.000

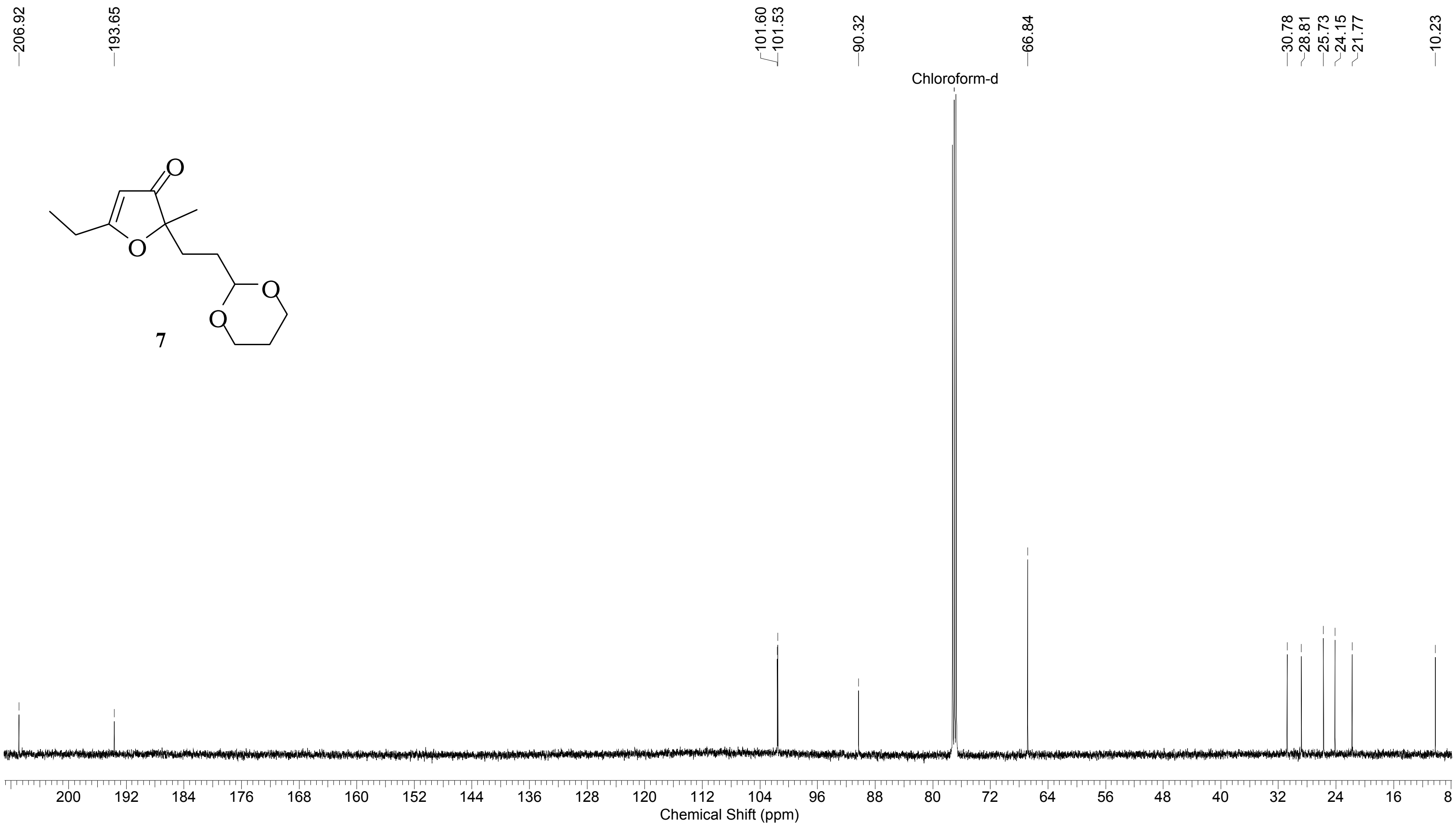

Figure S16. ^{13}C-NMR (DEPT 135) spectrum of compound **7** (125 MHz, $CDCl_3$).

Acquisition Time (sec)	1.0420	***Comment***	Imported from UXNMR.			***Date***	
File Name				***Frequency (MHz)***	125.76	***Nucleus***	13C
Number of Transients	512	***Original Points Count***	32768	***Points Count***	32768	***Pulse Sequence***	dept135
Solvent	CHLOROFORM-D			***Sweep Width (Hz)***	31446.54	***Temperature (degree C)***	27.000

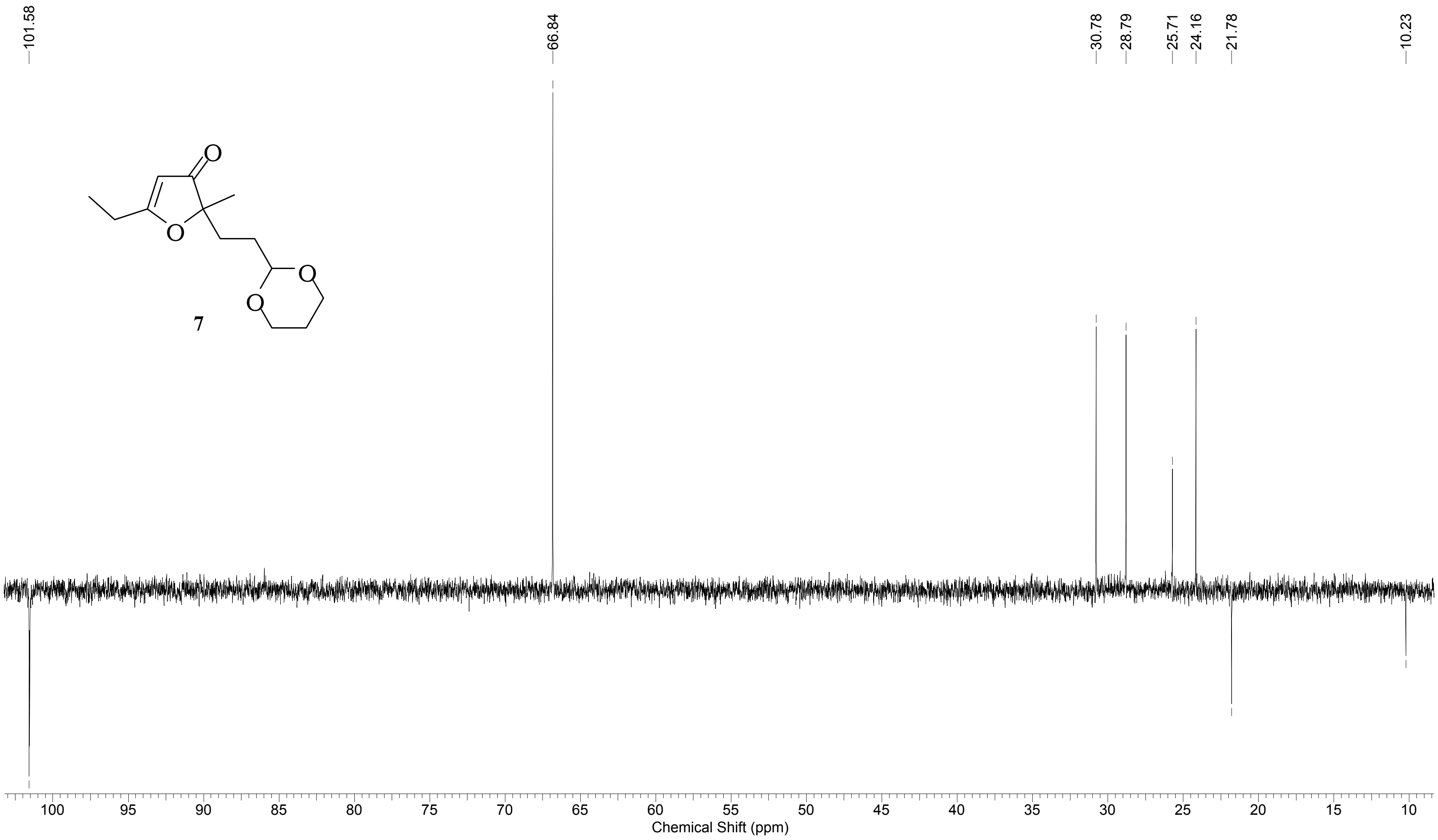

Figure S17. ^{1}H-NMR spectrum of compound **8** (500 MHz, $CDCl_3$).

Acquisition Time (sec)	3.8535	***Comment***	Imported from UXNMR.			***Date***			
File Name				***Frequency (MHz)***	500.13	***Nucleus***	1H	***Number of Transients***	16
Original Points Count	32768	***Points Count***	32768	***Pulse Sequence***	zg30	***Solvent***	CHLOROFORM-D		
Sweep Width (Hz)	8503.40	***Temperature (degree C)***	27.000						

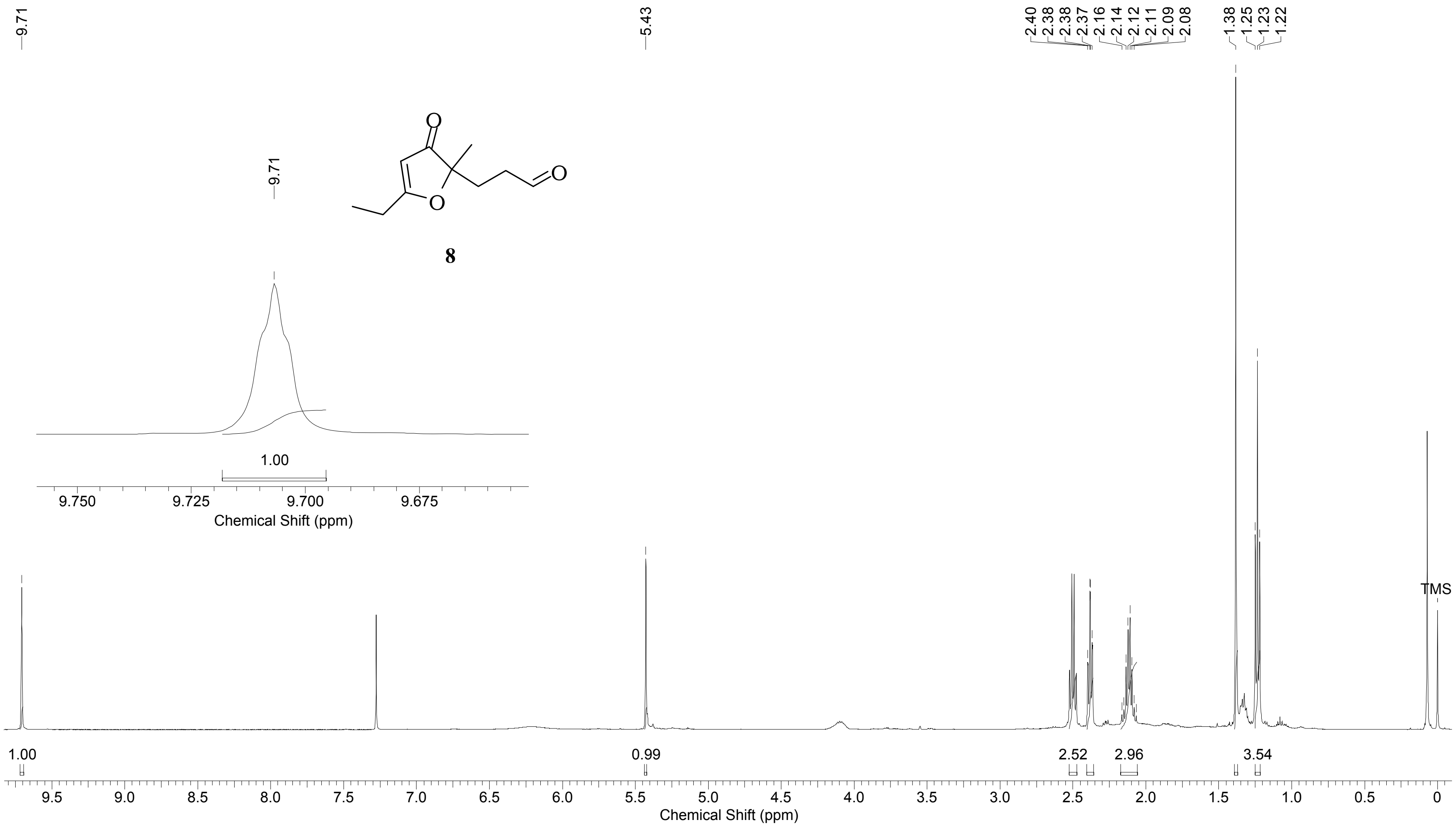

Figure S18. ^{13}C-NMR spectrum of compound **8** (125 MHz, $CDCl_3$).

Acquisition Time (sec)	1.0420	***Comment***	Imported from UXNMR.			***Date***	
File Name						***Frequency (MHz)***	125.76
Nucleus	13C	***Number of Transients***	3072	***Original Points Count***	32768	***Points Count***	32768
Pulse Sequence	zgpg30	***Solvent***	CHLOROFORM-D	***Sweep Width (Hz)***	31446.54	***Temperature (degree C)***	27.000

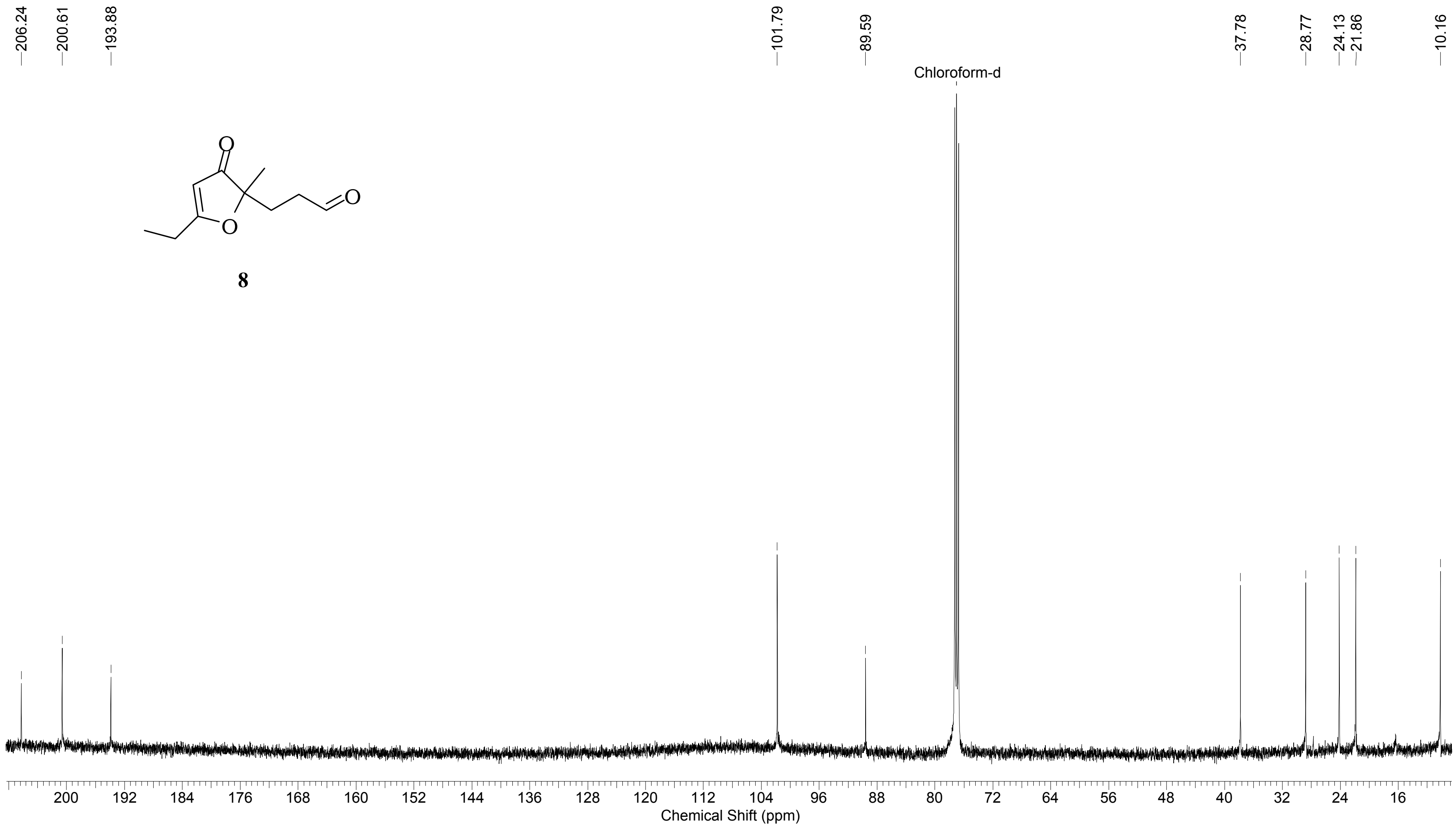

Figure S19. ^{13}C-NMR (DEPT 135) spectrum of compound **8** (125 MHz, $CDCl_3$).

Acquisition Time (sec)	1.0420	***Comment***	Imported from UXNMR.			***Date***	
File Name						***Frequency (MHz)***	125.76
Nucleus	13C	***Number of Transients***	1536	***Original Points Count***	32768	***Points Count***	32768
Pulse Sequence	dept135	***Solvent***	CHLOROFORM-D	***Sweep Width (Hz)***	31446.54	***Temperature (degree C)***	27.000

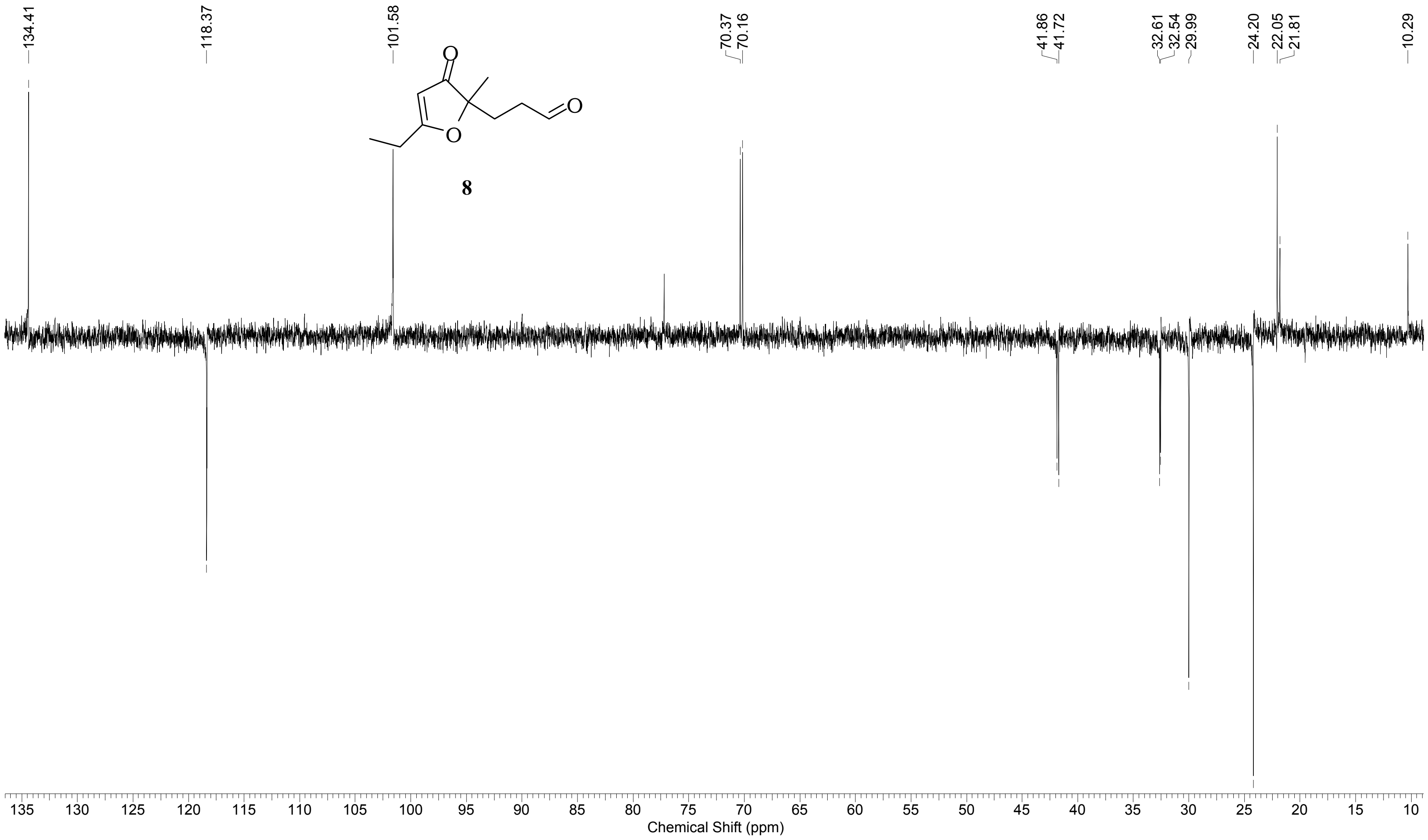

Figure S20. ¹H-NMR spectrum of compound **9** (500 MHz, $CDCl_3$).

Acquisition Time (sec)	3.8535	***Comment***	Imported from UXNMR.			***Date***	
File Name						***Frequency (MHz)***	500.13
Nucleus	1H	***Number of Transients***	16	***Original Points Count***	32768	***Points Count***	32768
Pulse Sequence	zg30	***Solvent***	CHLOROFORM-D			***Sweep Width (Hz)***	8503.40
Temperature (degree C)	27.000						

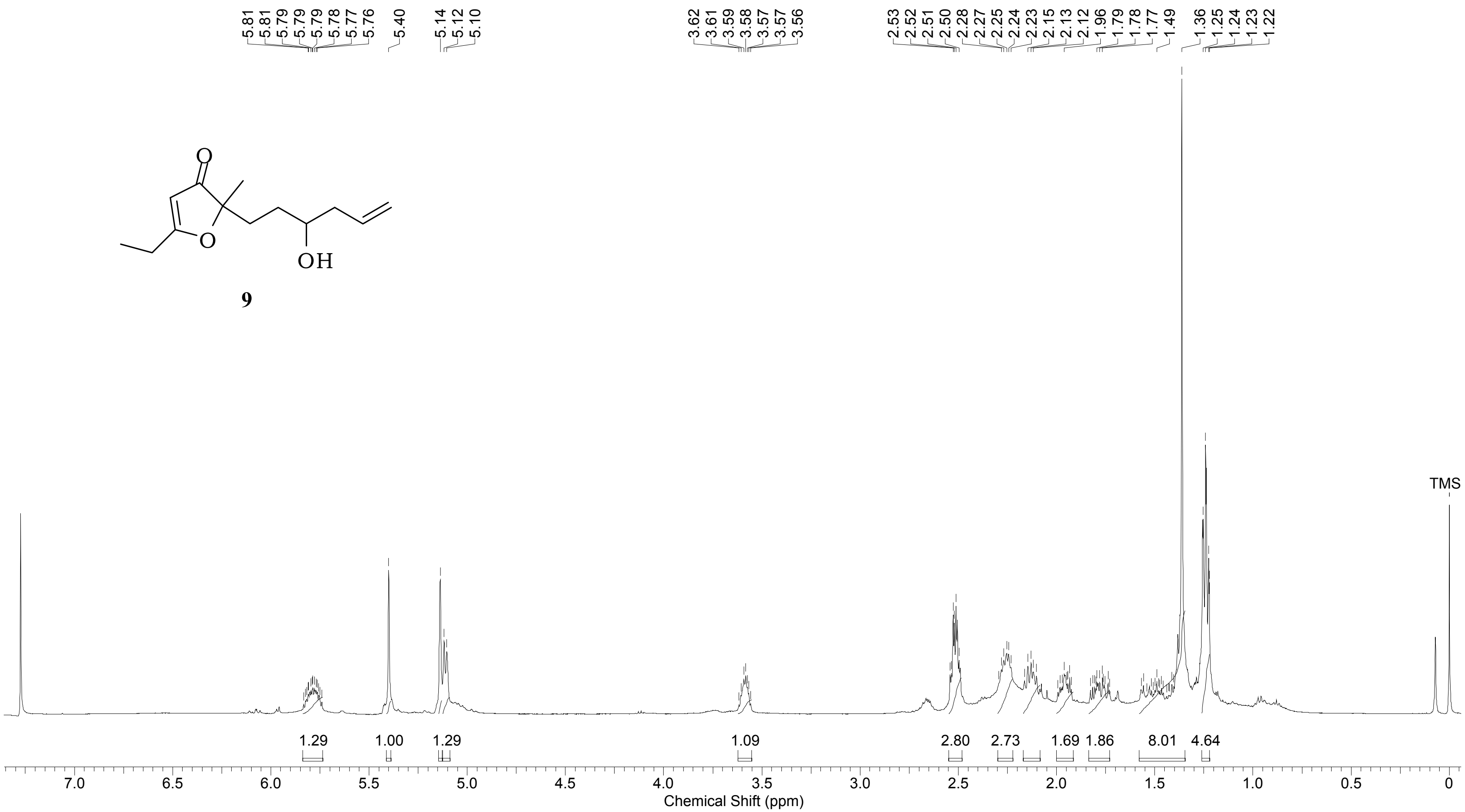

Figure S21. ^{13}C-NMR spectrum of compound **9** (125 MHz, $CDCl_3$).

Acquisition Time (sec)	1.0420	***Comment***	Imported from UXNMR.			***Date***	
File Name						***Frequency (MHz)***	125.76
Nucleus	13C	***Number of Transients***	3072	***Original Points Count***	32768	***Points Count***	32768
Pulse Sequence	zgpg30	***Solvent***	CHLOROFORM-D	***Sweep Width (Hz)***	31446.54	***Temperature (degree C)***	27.000

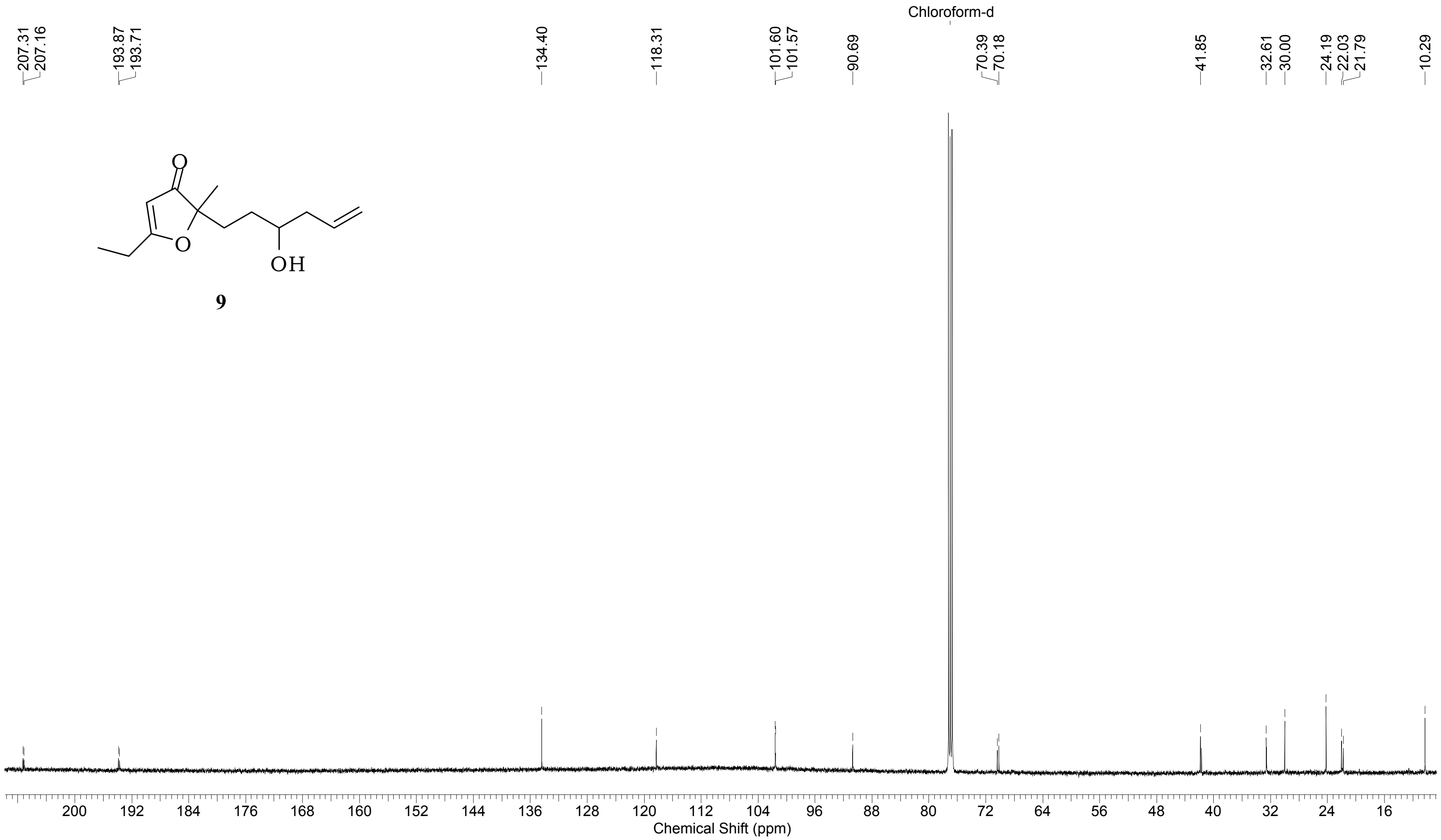

Figure S22. ^{13}C-NMR (DEPT 135) spectrum of compound **9** (125 MHz, $CDCl_3$).

Acquisition Time (sec)	1.0420	***Comment***	Imported from UXNMR.			***Date***	
File Name						***Frequency (MHz)***	125.76
Nucleus	13C	***Number of Transients***	1536	***Original Points Count***	32768	***Points Count***	32768
Pulse Sequence	dept135	***Solvent***	CHLOROFORM-D	***Sweep Width (Hz)***	31446.54	***Temperature (degree C)***	27.000

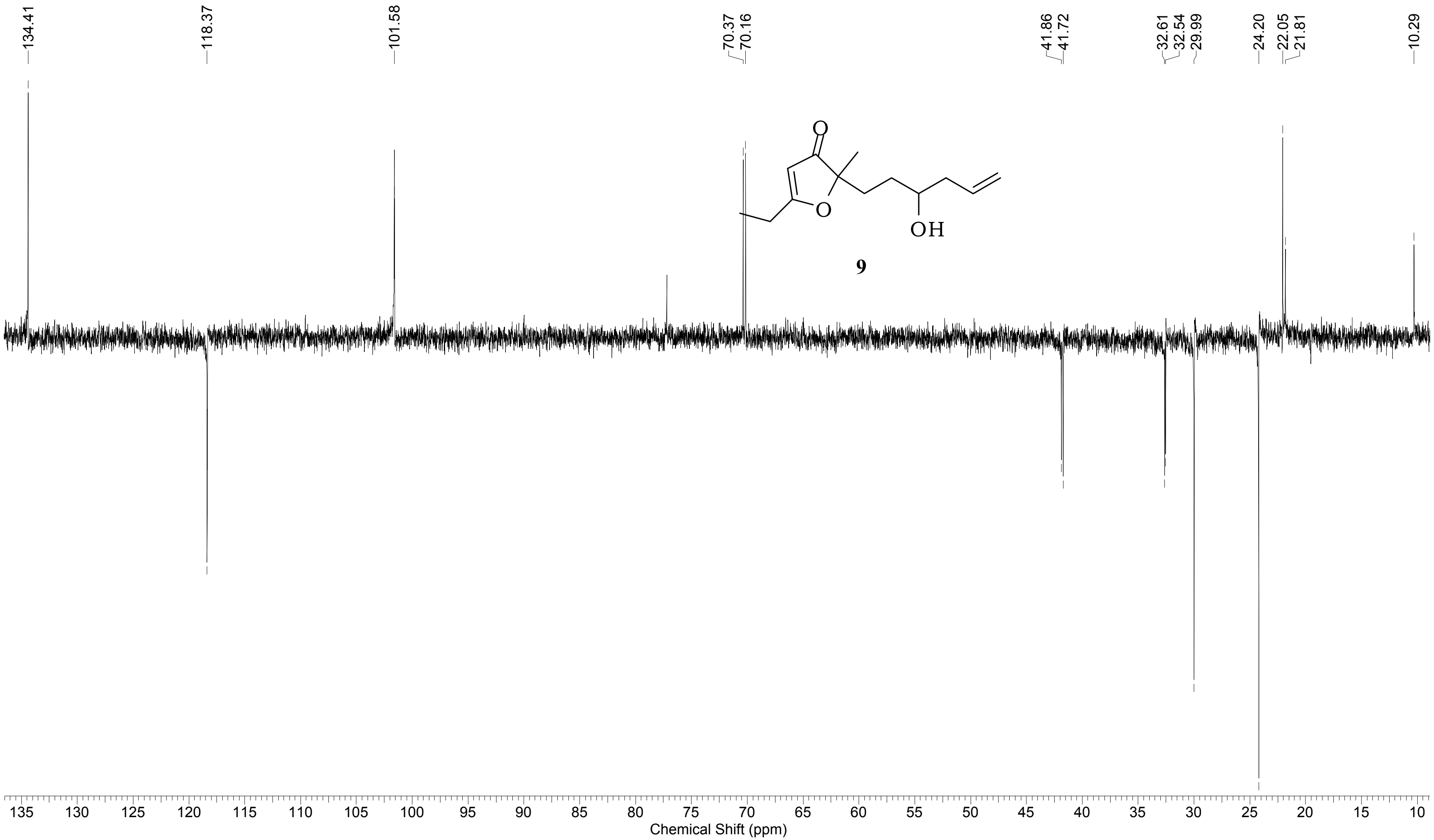

Figure S23. ^{1}H-NMR spectrum of compound **10** (500 MHz, $CDCl_3$).

Acquisition Time (sec)	6.0817	***Comment***	Imported from UXNMR.			***Date***	
File Name						***Frequency (MHz)***	500.13
Nucleus	1H	***Number of Transients***	64	***Original Points Count***	32768	***Points Count***	32768
Pulse Sequence	zg30	***Solvent***	CHLOROFORM-D			***Sweep Width (Hz)***	5387.93
Temperature (degree C)	27.000						

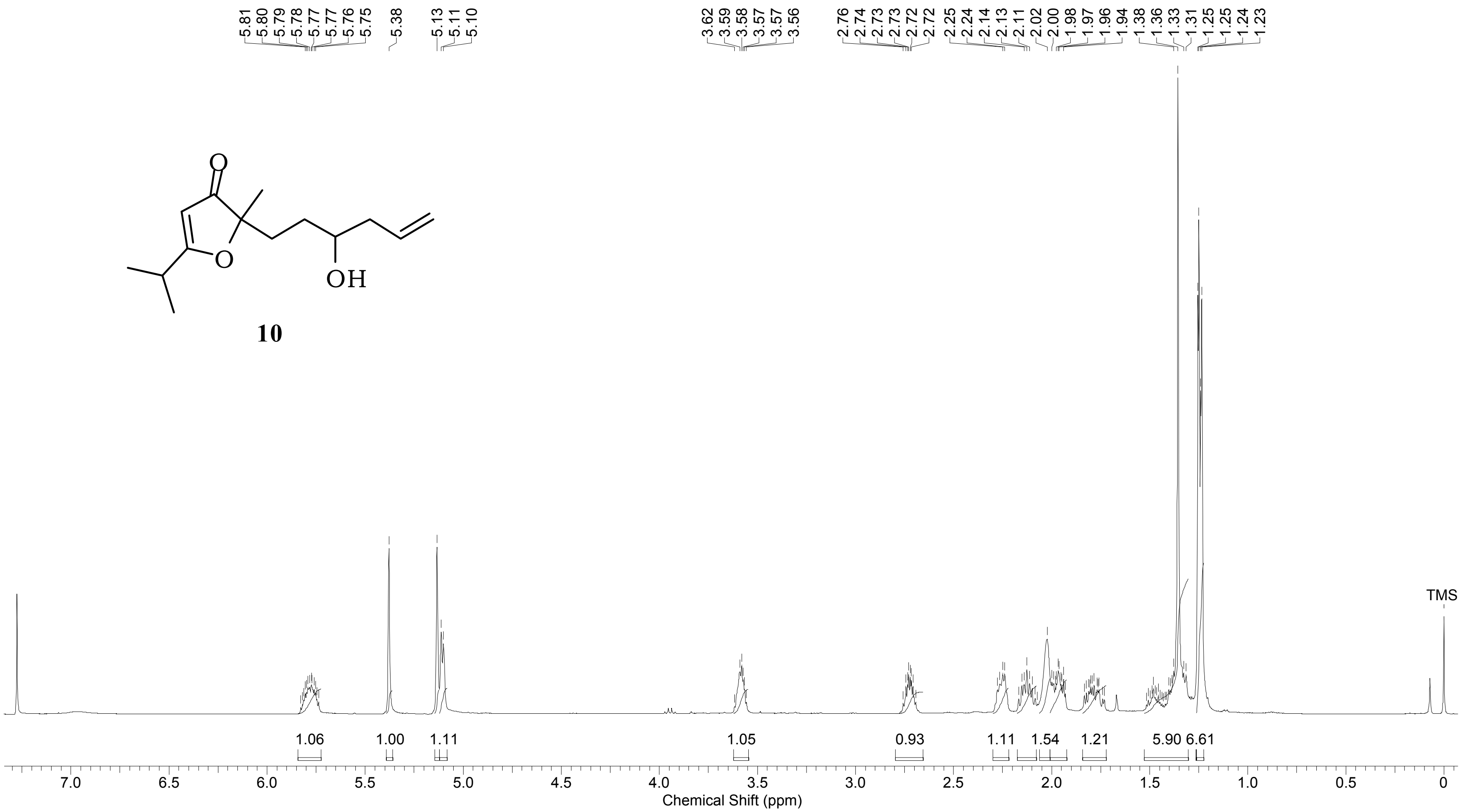

Figure S24. ^{13}C-NMR spectrum of compound **10** (125 MHz, $CDCl_3$).

Acquisition Time (sec)	1.0420	***Comment***	Imported from UXNMR.			***Date***	
File Name						***Frequency (MHz)***	125.76
Nucleus	13C	***Number of Transients***	3072	***Original Points Count***	32768	***Points Count***	32768
Pulse Sequence	zgpg30	***Solvent***	CHLOROFORM-D	***Sweep Width (Hz)***	31446.54	***Temperature (degree C)***	27.000

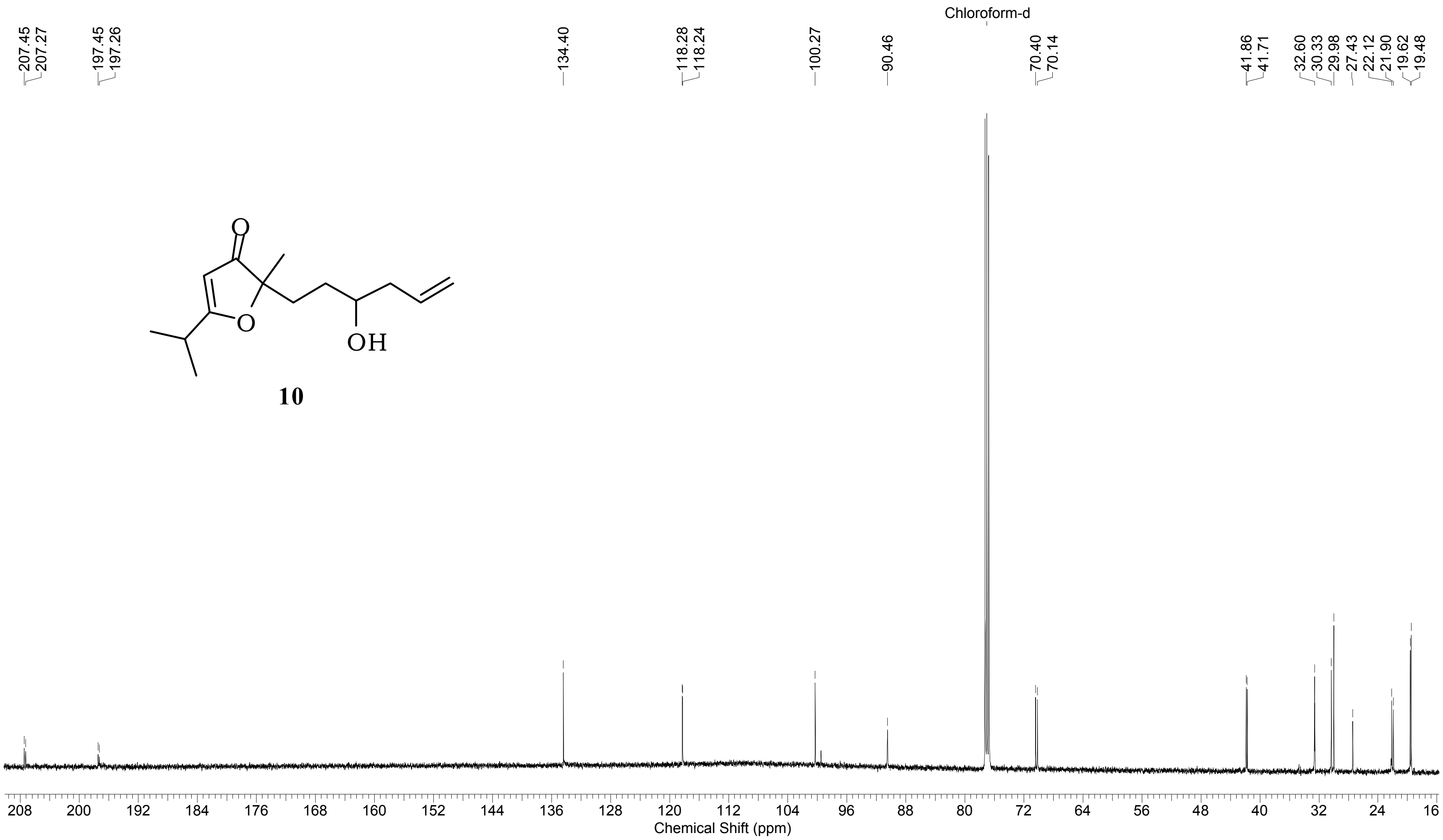

Figure S25. ^{13}C-NMR (DEPT 135) spectrum of compound **10** (125 MHz, $CDCl_3$).

Acquisition Time (sec)	1.0420	***Comment***	Imported from UXNMR.			***Date***	
File Name						***Frequency (MHz)***	125.76
Nucleus	13C	***Number of Transients***	1536	***Original Points Count***	32768	***Points Count***	32768
Pulse Sequence	dept135	***Solvent***	CHLOROFORM-D	***Sweep Width (Hz)***	31446.54	***Temperature (degree C)***	27.000

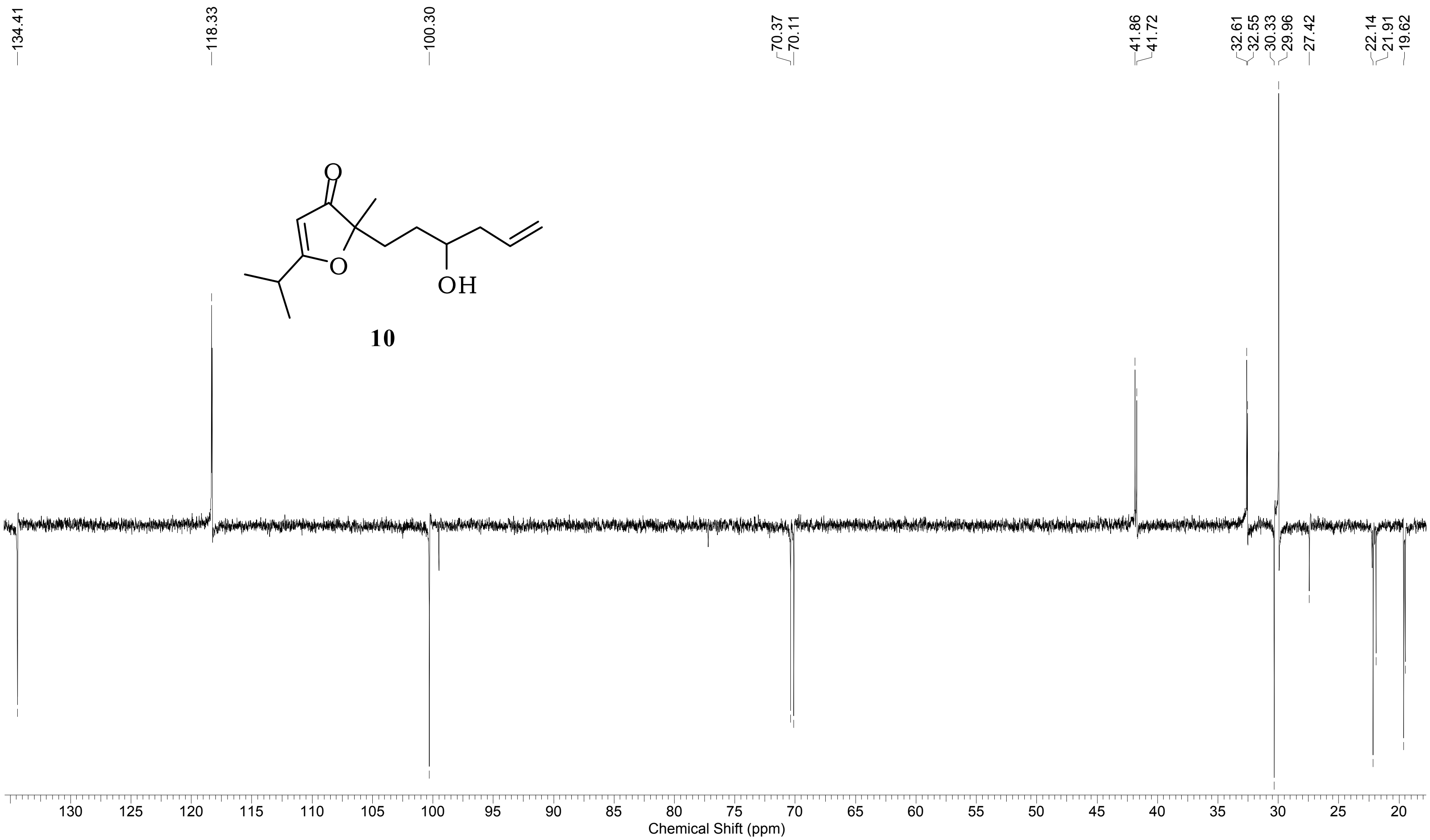

Figure S26. ^{1}H-NMR spectrum of compound **11** (500 MHz, $CDCl_3$).

Acquisition Time (sec)	3.8535	***Comment***	Imported from UXNMR.			***Date***	
File Name						***Frequency (MHz)***	500.13
Nucleus	1H	***Number of Transients***	16	***Original Points Count***	32768	***Points Count***	32768
Pulse Sequence	zg30	***Solvent***	CHLOROFORM-D			***Sweep Width (Hz)***	8503.40
Temperature (degree C)	27.000						

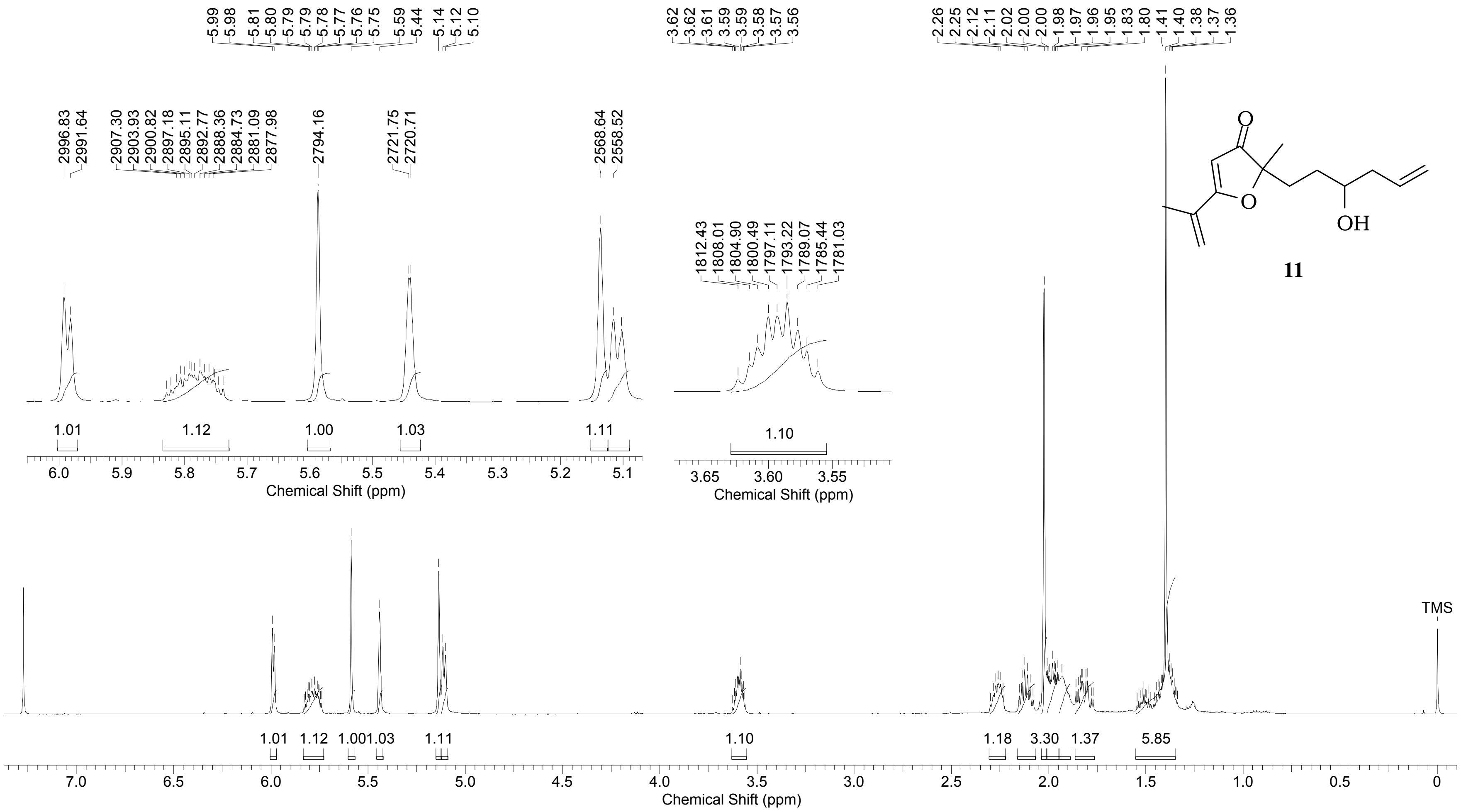

Figure S27. ^{13}C-NMR spectrum of compound **11** (125 MHz, $CDCl_3$).

Acquisition Time (sec)	1.0420	***Date***					
File Name						***Frequency (MHz)***	125.76
Nucleus	13C	***Number of Transients***	4096	***Original Points Count***	32768	***Points Count***	32768
Pulse Sequence	zgpg30	***Solvent***	CHLOROFORM-D			***Sweep Width (Hz)***	31446.54
Temperature (degree C)	27.000						

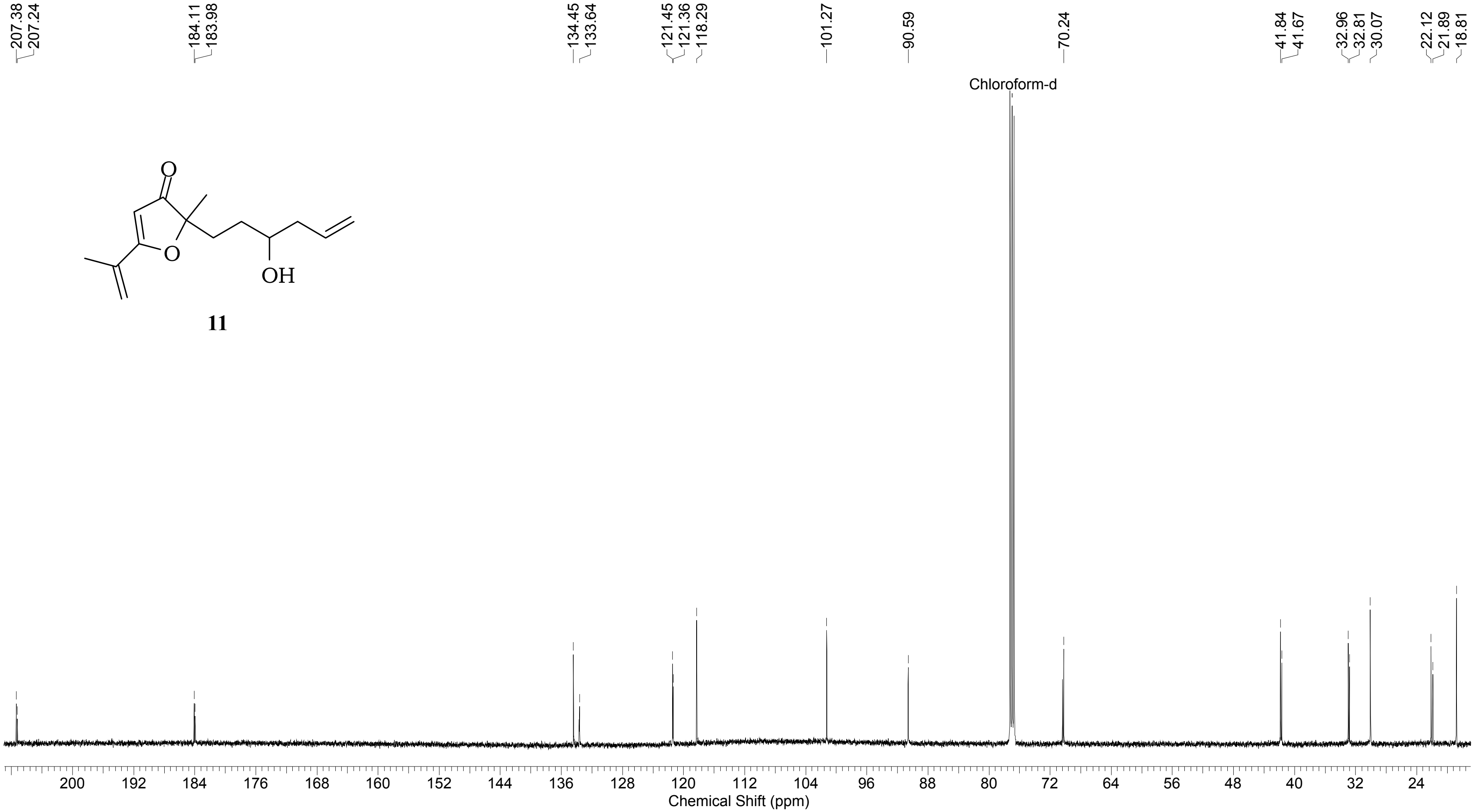

Figure S28. ^{13}C-NMR (DEPT 135) spectrum of compound **11** (125 MHz, $CDCl_3$).

Acquisition Time (sec)	1.0420	***Date***					
File Name						***Frequency (MHz)***	125.76
Nucleus	13C	***Number of Transients***	2048	***Original Points Count***	32768	***Points Count***	32768
Pulse Sequence	dept135	***Solvent***	CHLOROFORM-D			***Sweep Width (Hz)***	31446.54
Temperature (degree C)	27.000						

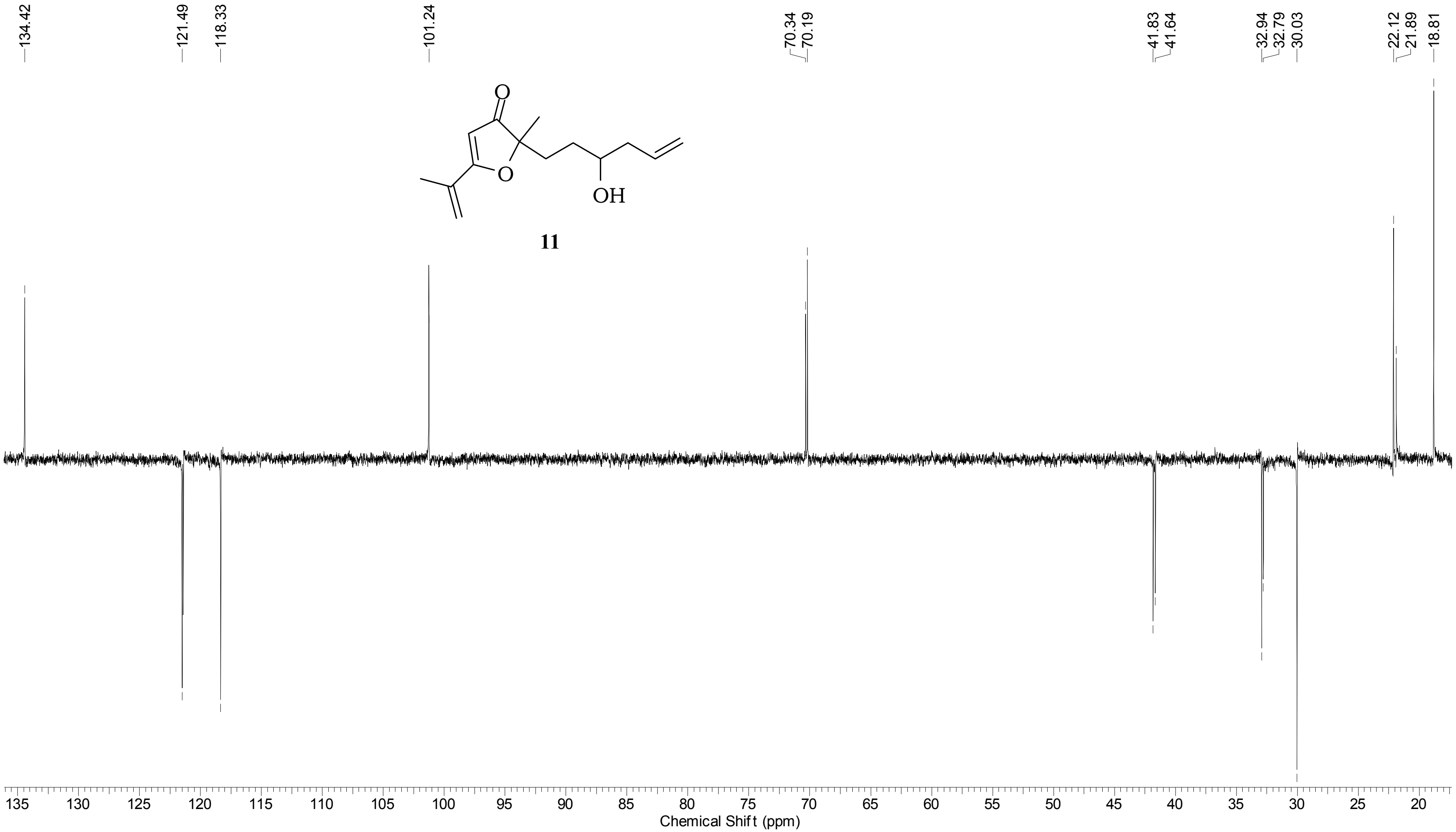

Supplement: Supplementary file 1 [file molecules-17-12151-s001.pdf]
